# ZNF460-mediated circRPPH1 promotes TNBC progression through ITGA5-induced FAK/PI3K/AKT activation in a ceRNA manner

**DOI:** 10.1186/s12943-024-01944-w

**Published:** 2024-02-14

**Authors:** Chuanpeng Zhang, Ziyi Yu, Susu Yang, Yitao Liu, Jiangni Song, Juan Mao, Minghui Li, Yi Zhao

**Affiliations:** Department of General Surgery, the First Affiliated Hospital of Nanjing Medical University, 300 Guangzhou Road, Nanjing, 210029 Jiangsu Province China

**Keywords:** TNBC, circRPPH1, miR-326, ITGA5

## Abstract

**Background:**

Circular RNAs are highly stable regulatory RNAs that have been increasingly associated with tumorigenesis and progression. However, the role of many circRNAs in triple-negative breast cancer (TNBC) and the related mechanisms have not been elucidated.

**Methods:**

In this study, we screened circRNAs with significant expression differences in the RNA sequencing datasets of TNBC and normal breast tissues and then detected the expression level of circRPPH1 by qRT‒PCR. The biological role of circRPPH1 in TNBC was then verified by in vivo and in vitro experiments. Mechanistically, we verified the regulatory effects between circRPPH1 and ZNF460 and between circRPPH1 and miR-326 by chromatin immunoprecipitation (ChIP), fluorescence in situ hybridization assay, dual luciferase reporter gene assay and RNA pull-down assay. In addition, to determine the expression of associated proteins, we performed immunohistochemistry, immunofluorescence, and western blotting.

**Results:**

The upregulation of circRPPH1 in TNBC was positively linked with a poor prognosis. Additionally, both in vivo and in vitro, circRPPH1 promoted the biologically malignant behavior of TNBC cells. Additionally, circRPPH1 may function as a molecular sponge for miR-326 to control integrin subunit alpha 5 (ITGA5) expression and activate the focal adhesion kinase (FAK)/PI3K/AKT pathway.

**Conclusion:**

Our research showed that ZNF460 could promote circRPPH1 expression and that the circRPPH1/miR-326/ITGA5 axis could activate the FAK/PI3K/AKT pathway to promote the progression of TNBC. Therefore, circRPPH1 can be used as a therapeutic or diagnostic target for TNBC.

**Supplementary Information:**

The online version contains supplementary material available at 10.1186/s12943-024-01944-w.

## Background

Breast cancer is one of the most prevalent malignant tumors in women and the main reason why women die from cancer [1]. Breast cancer of the TNBC kind lacks the ER, PR, and HER2 receptors. It makes up approximately 15% to 20% of all cases [2]. Due to the absence of expression of these receptors, chemotherapy continues to be the backbone of treatment [3]. However, TNBC is more prone to lymph node and even distant metastases [4]. Therefore, exploring new therapeutic targets for TNBC is necessary.

CircRNAs are a novel class of RNA molecules formed by head-to-back splicing of pre-mRNAs produced by host genes [5]. CircRNAs are stable because their circular structure is not easily degraded by RNA enzymes [6]. In addition, circRNAs can serve as ceRNAs to bind to miRNAs and thus affect the expression of downstream genes [7]. The abovementioned features of circRNAs will open up new possibilities and perspectives for cancer treatment and identification [8]. Numerous circRNAs have been demonstrated to have significant regulatory functions in the growth and progression of tumors. For instance, E2F1 and EIF4A3-induced circSEPT9 promotes TNBC initiation and progression [9]. Another circRNA, circMAPK1, which encodes MAPK1-109aa, can inhibit the progression of gastric cancer by preventing the MAPK pathway from becoming activated [10]. However, to date, there are fewer reports of circRNAs associated with TNBC.

MiRNAs are noncoding RNAs of approximately 23 nucleotides. They specifically target the mRNA 3' UTR, destabilizing it and inhibiting translation [11]. MiRNAs can either promote or inhibit tumor progression in a number of cancers. For instance, misregulation of Let-7 leads to reduced cell differentiation and cancer development [12]. MiR-10b is highly expressed in breast cancer and promotes metastasis and invasion of cancer cells [13]. These studies suggest that dysregulation of miRNA expression influences disease progression.

The ceRNA hypothesis proposes that RNAs with miRNA target sites can bind miRNAs in competition with mRNAs, forming a coregulatory posttranscriptional network [14]. Numerous studies imply that circRNAs can act as ceRNAs to regulate miRNAs [15–17]. Consequently, the imbalance of the ceRNA network may cause tumorigenesis and its development by disrupting the expression of key circRNAs. For instance, circUBXN7 binds miR-1247-3p and enhances B4GALT3 expression to hinder bladder cancer progression [18]. In addition, circCD44 obstructs miR-502-5p-induced KRAS degradation, hence encouraging TNBC proliferation, invasion, migration, and tumor formation [19]. However, the pathogenic and progression roles of many circRNAs in TNBC remain enigmatic.

Integrins act as cell surface receptors that regulate cell adhesion, cytoskeletal organization, signal transduction, and cell migration through the formation of focal adhesion complexes [20, 21]. ITGA5 is one of them. According to several studies, ITGA5 has oncogene-promoting functions and participates in the proliferation or metastasis of various malignant tumors [22, 23]. Further studies on the function of ITGA5 in TNBC are needed. FAK is a kinase that exists in the integrin family pathway [24, 25]. ITGA5 has been shown to interact with FAK and activate the phosphorylation of FAK, thereby activating the PI3K/AKT pathway [26, 27]. In addition, the PI3K/AKT pathway also causes the genesis and development of tumors [28, 29]. Therefore, it is crucial to research the relationship between the PI3K/AKT pathway and ITGA5 in TNBC.

The present study analyzed RNA sequencing data from GEO and found the differentially expressed circRNA circRPPH1 derived from RPPH1. We discovered that circRPPH1 expression is upregulated in TNBC tissues and cells, which was correlated with poor prognosis and TNM staging. We then demonstrated through cell function and animal experiments that circRPPH1 promotes TNBC cell growth and metastasis. In addition, it was discovered that the transcription factor ZNF460 can facilitate the formation of circRPPH1. Subsequent experiments showed that circRPPH1 could bind miR-326, thereby inhibiting the degradation of ITGA5, and was associated with the FAK/PI3K/AKT pathway, leading to the development of TNBC. In conclusion, circRPPH1 is a useful therapeutic and diagnostic target for TNBC.

## Methods

### TNBC tissue samples

Sixty pairs of tissues were obtained from patients with TNBC diagnosed at the First Affiliated Hospital of Nanjing Medical University. Each study participant signed an informed consent form. Our study was authorized by the First Affiliated Hospital of Nanjing Medical University's ethics committee.

### Cell culture

SUM1315 was generously provided by Stephen Ethier (University of Michigan, MI, USA). The normal breast cell line MCF10A, TNBC cell lines (MDA-MB-231, MDA-MB-468, BT549), and HEK-293 T cells were purchased from the American Type Culture Collection (ATCC, USA). High sugar DMEM or RPMI-1640 medium (Gibco, CA, USA) containing 1% penicillin and streptomycin (HyClone, UT, USA) and 10% fetal bovine serum (Biochannel, Nanjing, China) was used. The culture environment was 5% CO_2_ at 37 °C.

### Plasmid construction, RNAi and cell transfection

To knock down circRPPH1, siRNAs (si-circ1, si-circ2, si-circ3) targeting the reverse splice site of circRPPH1 and lentiviral vectors and control vectors containing shRNAs targeting circRPPH1 were purchased from GenePharma (Shanghai, China). The pCD5-ciR expression vector with the full-length sequence of human circRPPH1 inserted and a control mock vector without the circRPPH1 sequence were synthesized by GENESEED (Guangzhou, China) to overexpress circRPPH1. TNBC cells were transfected with circRPPH1 overexpression and mock vectors, lentiviral vectors containing sh-NC and sh-circ, and screened with puromycin to establish cell lines stably overexpressing or knocking down circRPPH1. MiR-326 mimics and inhibitors and knockdown plasmids (sh1-ZNF460, sh2-ZNF460, sh3-ZNF460) were synthesized by RiboBio (Nanjing, China). The pcDNA3.1-ZNF460 overexpression vectors and ZNF460 negative control vectors were acquired from Sangon Biotech (Shanghai, China). Lipofectamine 2000 (Invitrogen, Carlsbad, CA, USA) was used for transfection in accordance with the instructions. Transfection efficiency was determined after 48 h. Relevant sequences are shown in Additional file 1: Table S1.

### Animal experiments

Four-week-old female BALB/c nude mice were purchased from Nanjing Medical University's laboratory animal center. Subcutaneous injections of 1 × 10^6^ transfected cells that were either overexpressed or knocked down in circRPPH1 were given to naked mice. Tumor volume was measured weekly (volume = length × width^2^ × 0.5), and the tumor and lung were removed for analysis after 4 weeks. We injected different groups of cell lines transfected with luciferase-labeled cells into the tail vein of nude mice and observed distant metastases 3 weeks later using bioluminescence imaging. The Nanjing Medical University Ethics Committee gave its approval to each trial.

### Statistical analyses

ANOVA and Student's t test were used to evaluate the comparison between the groups. Clinicopathologic characterization was performed using the chi-square test. Correlation was analyzed using linear regression or Pearson analysis. The Kaplan‒Meier method was used for the survival study. ROC curves have been used to evaluate diagnostic value. Data are expressed as the mean ± standard deviation (SD). Statistical significance was defined as *p < 0.05, **p < 0.01, and ***p < 0.001. All experiments were performed at least 3 times.

## Results

### Identification and clinical characterization of circRPPH1

**Fig. 1:**
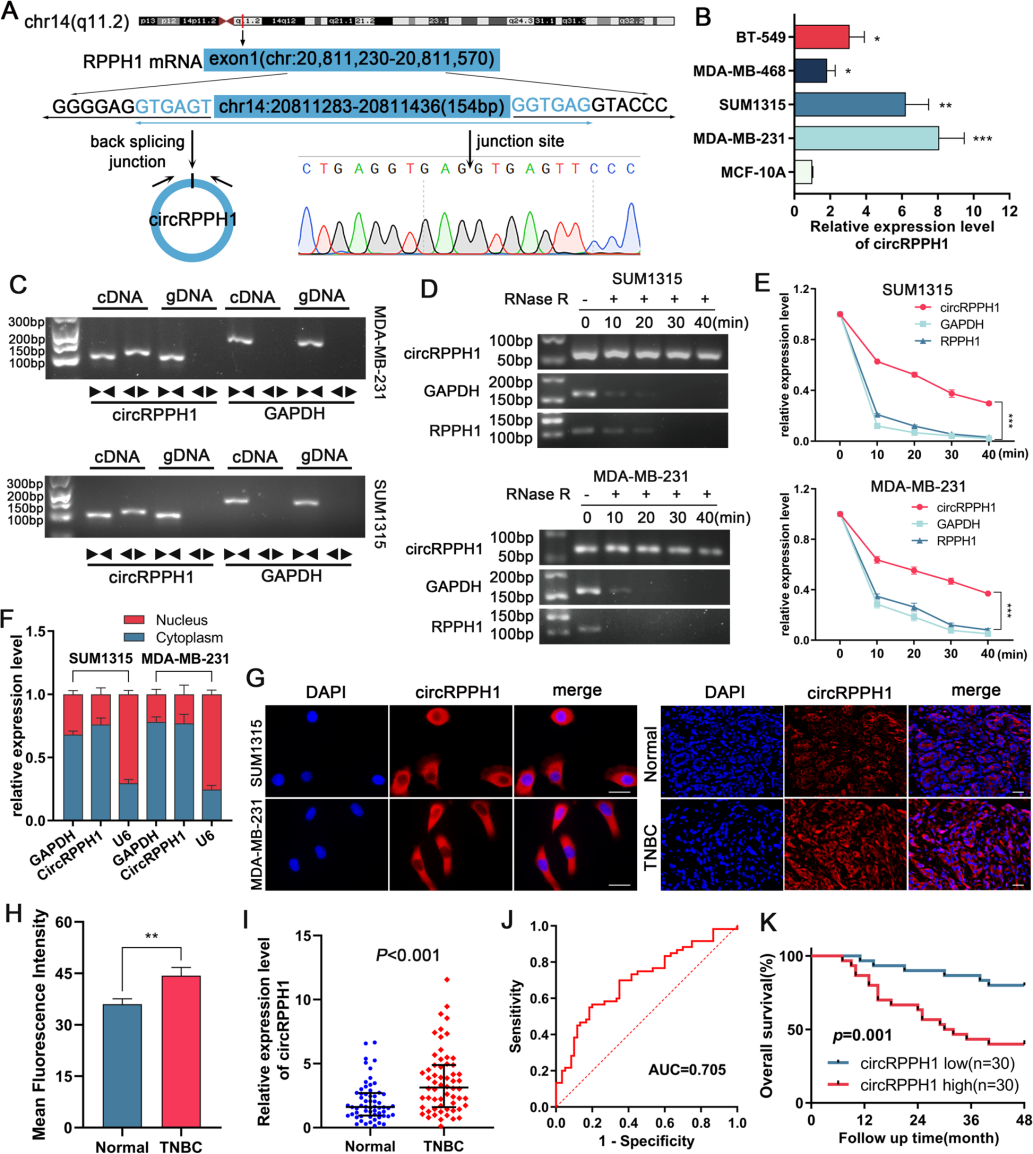
Identification and expression of circRPPH1 in TNBC. **a** Schematic diagram of circRPPH1 cyclization, and Sanger sequencing verified the backsplicing sequence and cyclization site of circRPPH1. **b** Relative expression levels of circRPPH1 in TNBC cell lines were analyzed by qRT-PCR. **c** Divergent primers detected circRPPH1 in cDNA but not in gDNA, and GAPDH was used as a negative control. **d** and **e** TNBC cells were analyzed for the abundance of RNase R-treated circRPPH1 and linear RPPH1 mRNA at the indicated time points. **f** Nucleo-plasmic separation experiments showed that circRPPH1 was predominantly localized in the cytoplasm of TNBC cells. GAPDH and U6 were used as internal references for cytoplasmic and nuclear transcripts, respectively. **g** FISH was performed to detect the localization of circRPPH1 in TNBC tissues (magnification, × 100, scale bar, 50 μm) and cells (magnification, × 200, scale bar, 20 μm). DAPI stained nuclei. **h** Mean fluorescence intensity analysis of TNBC tissue and normal breast tissue in FISH assay. **i** The relative expression levels of circRPPH1 in 60 pairs of TNBC tissues were analyzed by qRT-PCR. **j** The diagnostic value of circRPPH1 for TNBC was evaluated using ROC curves (cut-off value:0.367). **k** Overall survival analysis of 60 TNBC patients based on circRPPH1 expression

We filtered out 20 circRNAs with the most notable expression changes by examining the RNA sequencing arrays of TNBC tissue and normal breast tissue from the GSE101123 dataset (Supplementary Fig. 1A and B). The relationship between hsa_circRNA_000166 and TNBC is unknown among the identified circRNAs. This work will focus on its biological activities and associated mechanisms in TNBC. Hsa_circRNA_000166 (referred to as circRPPH1 in this study) is generated from the mRNA encoded by the RPPH1 gene by backward splicing, and Sanger sequencing proved the existence of its backward splice site (Fig. 1A). We then verified by qRT‒PCR that circRPPH1 expression was markedly upregulated in SUM1315 and MDA-MB-231 cells compared to MCF-10A cells (Fig. 1B). To verify that circRPPH1 is a circular head-to-tail junction, we extracted cDNA and gDNA from TNBC cells and then used convergent primers and divergent primers for PCR amplification. The outcomes demonstrated that circRPPH1 may be amplified from cDNA rather than gDNA utilizing divergent primers. This indicates that circRPPH1 is a circular structure and is generated by posttranscriptional shearing of head-to-tail junctions (Fig. 1C). The RNase R assay illustrated that circRPPH1 exhibits higher resistance to degradation than the parental gene and GAPDH. This suggests that circRPPH1 is a more stable circular RNA than linear RNAs (Fig. 1D and E). Nuclear-cytoplasmic separation and FISH assays revealed the major presence of circRPPH1 in the cytoplasm, and the mean fluorescence intensity showed that TNBC tissues expressed more circRPPH1 than normal breast tissues (Fig. 1F-H). To explore the application value of circRPPH1, we detected increased expression of circRPPH1 in TNBC tissues (Fig. 1I). The ROC curve showed that the specificity was 0.8, the sensitivity was 0.567, and the area under the curve was 0.705 (Fig. 1J). We divided TNBC patients into two groups based on median circRPPH1 expression to assess the relationship between clinicopathological features and high and low circRPPH1 expression. The findings demonstrated that whereas menopausal status, grade, and other clinicopathological factors were not connected with circRPPH1 expression, they had a positive relationship with the T, N, and TNM stages (Table 1). We subsequently plotted Kaplan‒Meier survival curves based on TNBC patient follow-up data, which revealed that patients who had higher circRPPH1 expression had a shorter overall survival than patients who had lower circRPPH1 expression (Fig. 1K). High circRPPH1 expression was discovered by Cox regression models to be a standalone predictor of overall survival in TNBC patients (Supplementary Fig. 1C and D). According to these findings, circRPPH1 can be utilized for both diagnosis and prognosis.

**Table 1:** Correlation between circRPPH1 expression and clinicopathological features in 60 TNBC patients

Characteristics		CircRPPH1	*P* value	
		Low	High	
		30	30	
Age	≥ 50	12 (20%)	13 (21.7%)	0.793
	< 50	18 (30%)	17 (28.3%)	
Menopausal	Postmenopausal	11 (18.3%)	12 (20%)	0.791
	Premenopausal	19 (31.7%)	18 (30%)	
Grage	II	18 (30%)	12 (20%)	0.121
	III	12 (20%)	18 (30%)	
T stage	T1	18 (30%)	7 (11.7%)	0.004**
	T2-3	12 (20%)	23 (38.3%)	
N stage	N0	21 (35%)	13 (21.7%)	0.037*
	N1-3	9 (15%)	17 (28.3%)	
TNM stage	I	14 (23.3%)	3 (5%)	0.002**
	II/III	16 (26.7%)	27 (45%)	
Vascular invasion	No	21 (35%)	18 (30%)	0.417
	Yes	9 (15%)	12 (20%)	

### ZNF460 increases the expression of RPPH1 and circRPPH1

**Fig. 2:**
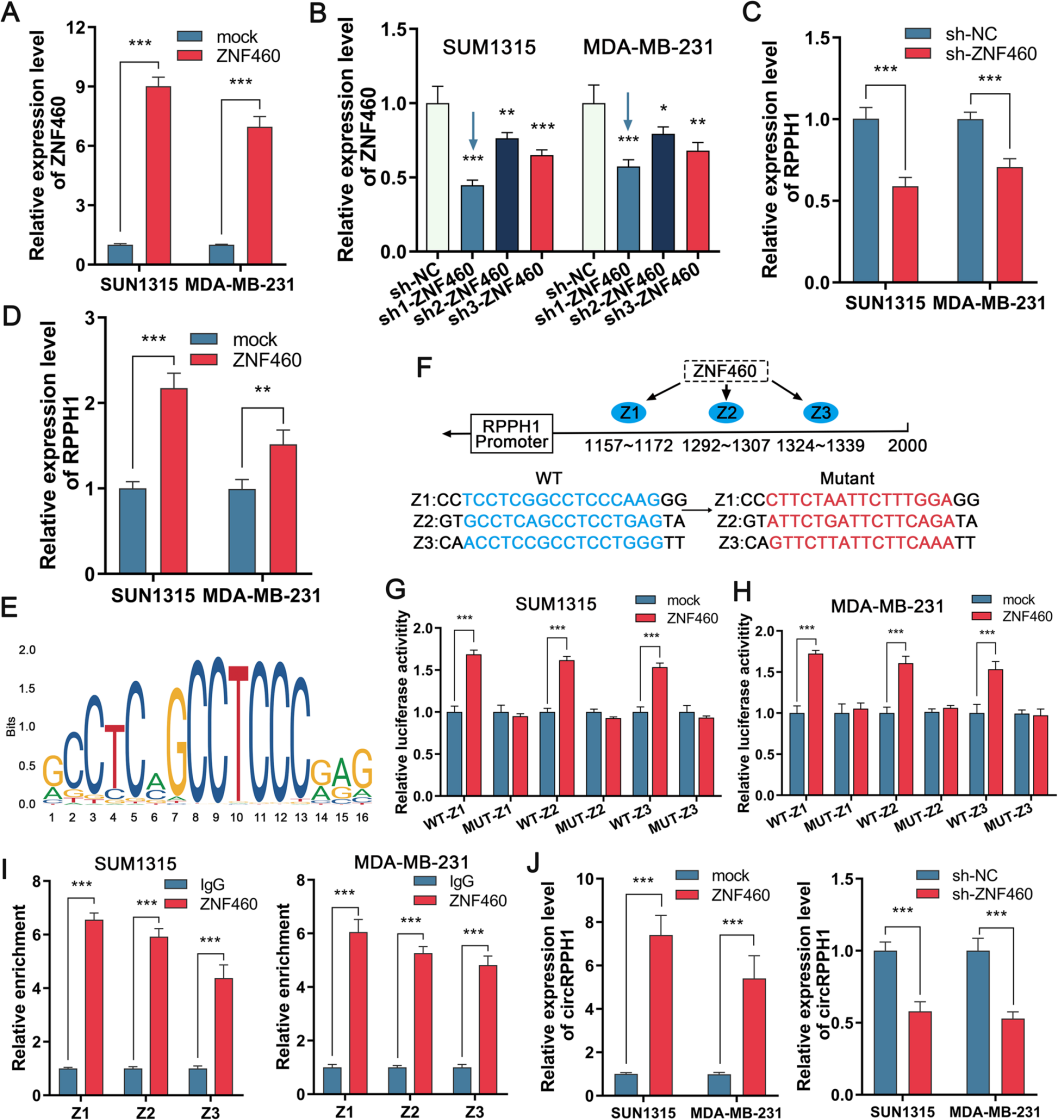
ZNF460 increases circRPPH1 expression in TNBC cells. **a** and **b** Detection of ZNF460 expression in TNBC cells after transfection with ZNF460 overexpression plasmid or siRNA. **c** and **d** Detection of RPPH1 expression in TNBC cells after overexpression or knockdown of ZNF460 by qRT-PCR. **e** Plot of base frequency analysis of the binding site of ZNF460 to the RPPH1 promoter. **f** Schematic representation of the wild-type (WT) and mutant (Mut) sequences of the three predicted binding sites on the promoter of ZNF460 and RPPH1. **g** and **h** Relative luciferase activities were determined after co-transfection of TNBC cells with luciferase reporter plasmids containing wild-type or mutant RPPH1 promoter sequences and ZNF460 overexpression plasmids. **i** ChIP-qPCR assay to verify the binding site of the RPPH1 promoter to ZNF460 in TNBC cells, IgG was used as a negative control. **j** TNBC cells were analyzed for circRPPH1 expression after transfection with ZNF460 overexpression plasmid or siRNA

To explore whether there are transcription factors affecting the expression of RPPH1 and circRPPH1, we looked up the possible sequences of the RPPH1 promoter by NCBI (http://genome.ucsc.edu/) and then found three binding sites of the transcription factor ZNF460 with the RPPH1 promoter in the JASPAR database (https://jaspar.genereg.net/). We hypothesized that ZNF460 may impact the transcriptional regulation of RPPH1 expression. To further investigate the role of ZNF460, we designed overexpression vectors (pcDNA3.1-ZNF460) and knockdown vectors (sh1-ZNF460, sh2-ZNF460, sh3-ZNF460) and corresponding negative control vectors of ZNF460 and verified their effective expression by qRT‒PCR (Fig. 2A and B). The expression of RPPH1 was then greatly reduced after transfection with sh1-ZNF460 in TNBC cells, but it was significantly elevated following transfection with the overexpression vector pcDNA3.1-ZNF460 (Fig. 2C and D). According to the binding sequence of ZNF460 and the RPPH1 promoter, we constructed luciferase reporter plasmids with wild-type or mutant RPPH1 promoter sequences (Fig. 2E and F). The findings revealed that ZNF460 enhanced the luciferase activity of the wild-type RPPH1 promoter plasmids, whereas the mutant plasmids remained the same (Fig. 2G and H). ZNF460 was shown to bind to the RPPH1 promoter by the ChIP‒qPCR experiment, in contrast to the negative control IgG (Fig. 2I). Finally, we verified by RT‒qPCR that intracellular circRPPH1 expression was increased after transfection of pcDNA3.1-ZNF460 in TNBC cells, while circRPPH1 expression was decreased after transfection of sh1-ZNF460 (Fig. 2J). According to the aforementioned findings, ZNF460 may attach to the RPPH1 promoter region, thereby regulating its and circRPPH1 expression.

### CircRPPH1 promotes TNBC cell growth, migration and invasion

**Fig. 3:**
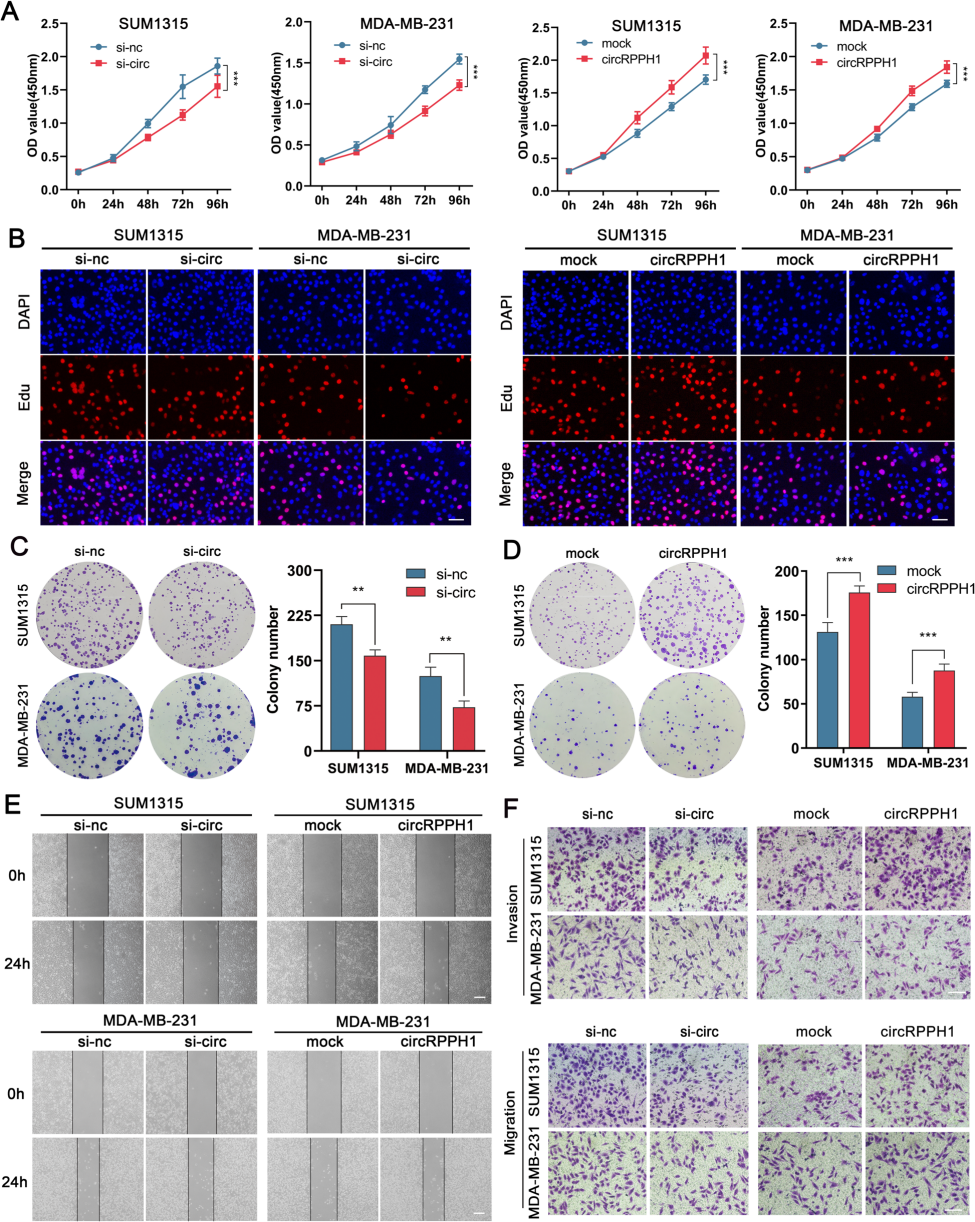
circRPPH1 promotes TNBC cell proliferation, migration and invasion. **a-d** Proliferation of TNBC cells after knockdown or overexpression of circRPPH1 was detected by CCK8, EDU assay (magnification, × 100, scale bar, 50 μm) and colony formation assay. **e** The effect of circRPPH1 on the migration ability of TNBC cells was detected by wound healing assay (magnification, × 40, scale bar, 100 μm). **f** The effect of circRPPH1 on the migration and invasion ability of TNBC cells was assessed by Transwell assay (magnification, × 100, scale bar, 100 μm)

To increase or knock down circRPPH1 expression, we designed overexpression vectors and small interfering RNAs based on the sequence and cyclization sites of circRPPH1, respectively (Supplementary Fig. 2A). Data from qRT‒PCR demonstrated that transfection of overexpression vectors or siRNA segments into TNBC cells greatly increased or decreased the expression of circRPPH1, respectively (Supplementary Fig. 2B and C). Si-circ1 was knocked down most efficiently in TNBC cells and was therefore used for subsequent experiments. However, circRPPH1 was overexpressed or knocked down with no significant effect on the parental gene RPPH1 (Supplementary Fig. 2D). We verified that knockdown of circRPPH1 inhibited the proliferation of TNBC cells, whereas overexpression of circRPPH1 promoted cell proliferation by colony formation assay, EdU and CCK-8 assay (Fig. 3A-D). In addition, Transwell and wound healing experiments showed that interfering with circRPPH1 could reduce the migration and invasion ability of TNBC cells, while overexpression of circRPPH1 did the opposite (Fig. 3E and F). According to the aforementioned findings, circRPPH1 exerted a pro-carcinogenic role in TNBC cells.

### CircRPPH1 promotes TNBC cell EMT, regulates the cell cycle and inhibits apoptosis

**Fig. 4:**
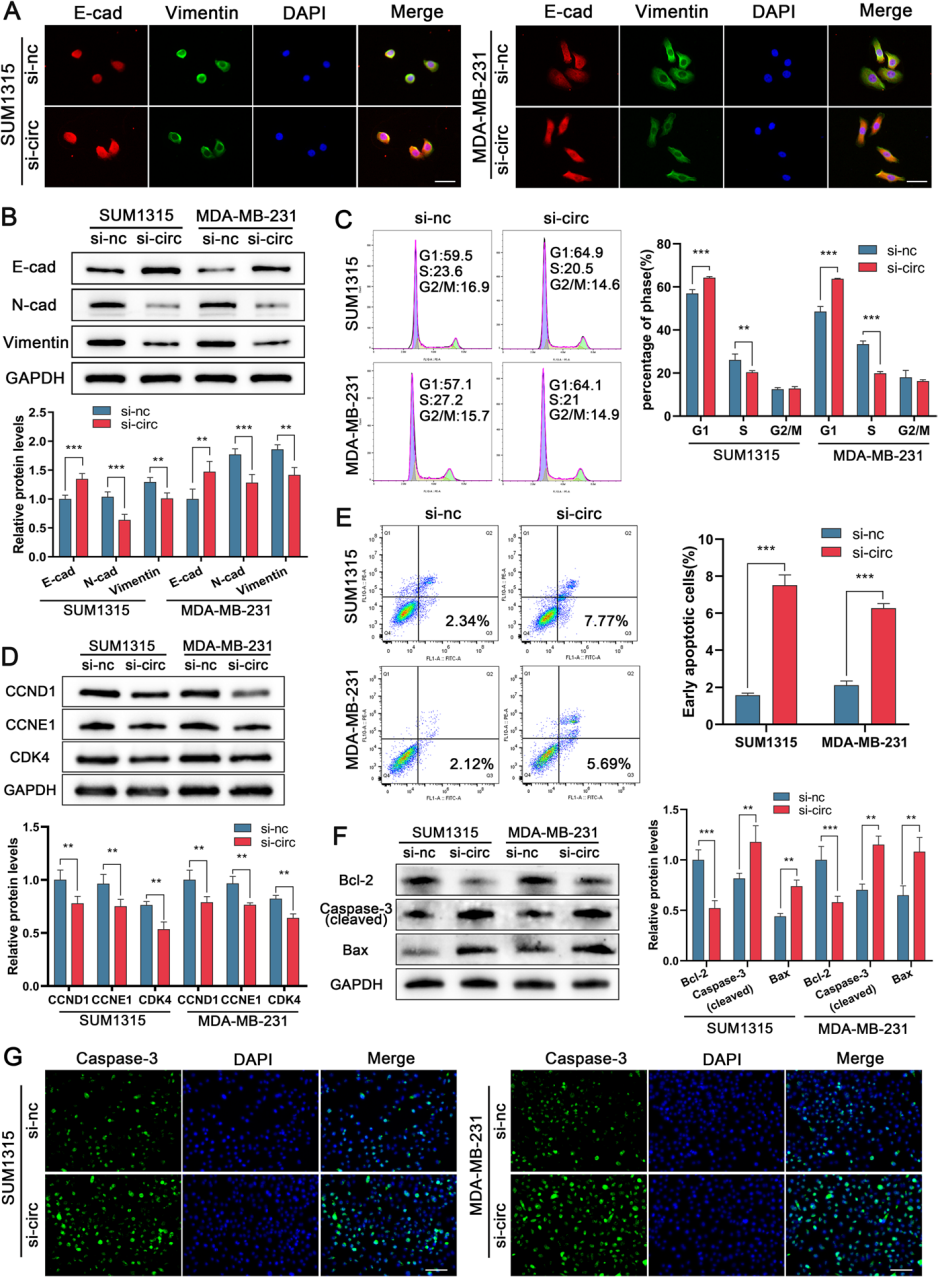
Knockdown of circRPPH1 inhibits EMT and regulates cell cycle and apoptosis in TNBC cells. **a** IF detection of EMT-related protein expression in TNBC cells after knockdown of circRPPH1 (magnification, × 200, scale bar, 50 μm). **b** Western blot detection of EMT-related protein expression in TNBC cells after knockdown of circRPPH1.**c** Cell cycle analysis of TNBC cells transfected with si-circRPPH1 by flow cytometry. **d** Western blot detection of cell cycle-related protein expression after transfection of si-circRPPH1 in TNBC cells. **e** Early apoptosis rate of TNBC cells was detected by flow cytometry after knockdown of circRPPH1. **f** Western blot detection of apoptosis-related protein expression. **g** IF detection of caspase-3 expression in TNBC cells after knockdown of circRPPH1 (magnification, × 200, scale bar, 100 μm)

Given that the EMT mechanism also contributes to tumor cell metastasis, we utilized an immunofluorescence assay to determine the levels of related proteins. Our findings revealed that after silencing circRPPH1, E-cadherin expression increased in TNBC cells, while N-cadherin and vimentin decreased (Fig. 4A). Moreover, the outcomes of Western blot experiments were consistent with the IF results (Fig. 4B). Next, we used flow cytometry for cell cycle detection, and our data revealed that after silencing circRPPH1, the proportion of TNBC cells remaining in G1 phase increased, and that in S phase decreased (Fig. 4C). We also detected a decrease in cell cycle-related protein expression after silencing circRPPH1 by Western blot (Fig. 4D). This suggests that the cell cycle is blocked after silencing circRPPH1. After that, we carried out an Annexin V/PI staining flow cytometry study and discovered that circRPPH1 knockdown greatly improved the early apoptotic rate of the cells (Fig. 4E). Similarly, we discovered using Western blot that after circRPPH1 knockdown, Bcl-2 expression was reduced, whereas cleaved caspase-3 and Bax expression were elevated (Fig. 4F). The above experiments showed opposite results after overexpression of circRPPH1 (Supplementary Fig. 3). In addition, immunofluorescence experiments showed that caspase-3 expression increased after knockdown of circRPPH1 (Fig. 4G). In conclusion, circRPPH1 has the biological functions of promoting EMT, accelerating the cell cycle and inhibiting apoptosis in TNBC cells.

### CircRPPH1 serves as a molecular sponge by directly binding to miR-326

**Fig. 5:**
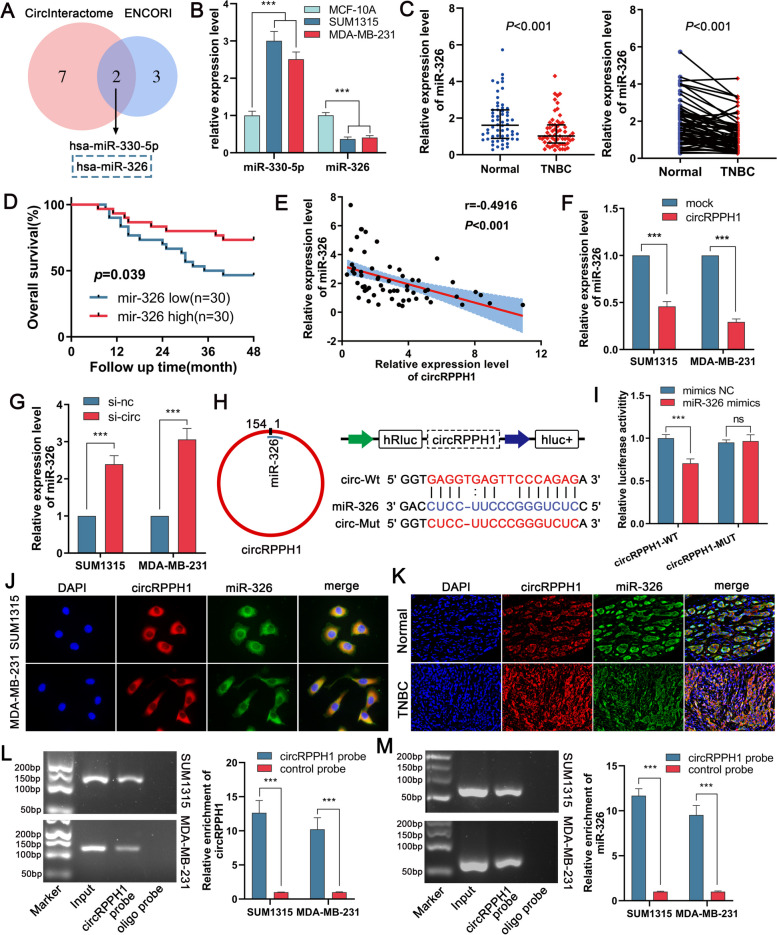
circRPPH1 binds to miR-326 and acts as its molecular sponge. **a** Venn diagram showing the intersection of CircInteractome and ENCORI database circRPPH1 target miRNAs. **b** Relative expression of miR-330-5p and miR-326 in cell lines. **c** Relative expression of miR-326 in 60 pairs of TNBC tissues and adjacent normal tissues. **d** Overall survival curves were plotted based on miR-326 expression in 60 pairs of TNBC patients. **e** Pearson correlation analysis showed that miR-326 was negatively correlated with circRPPH1 expression in 60 pairs of TNBC tissues. **f** and **g** The relative expression of miR-326 was detected after overexpression or knockdown of circRPPH1 in TNBC cells. **h** Schematic diagrams of the binding sites of circRPPH1 and miR-326 and the circRPPH1-Wt and circRPPH1-Mut luciferase reporter vectors. **i** Relative activities of luciferase in 293 T cells were assayed after transfection of circRPPH1-Wt or circRPPH1-Mut and miR-326 mimics or mimics NC, respectively. **J** and **k** FISH experiments showed co-localization of circRPPH1 with miR-326 in tumor cells (magnification × 100, scale bar, 50 μm) and cells (magnification, × 200, scale bar, 20 μm). **l** and **m** RNA pull-down in TNBC cells with a biotin-labeled circRPPH1 probe followed by detection of circRPPH1 and miR-326 enrichment

CircRNAs are known to operate as molecular sponges to regulate microRNAs, which have an impact on target genes downstream. To learn more about how circRPPH1 functions in TNBC, we predicted that hsa-miR-330-5p and hsa-miR-326 might interact with circRPPH1 using the ENCORI database (https://rnasysu.com/encori/) and CircInteractome database (https://circinteractome.nia.nih.gov/) (Fig. 5A). According to the TCGA database, the expression of hsa-miR-326 was downregulated in TNBC, and the prognosis was better in the low-expression group, while the expression of hsa-miR-330-5p was upregulated, which had no significant effect on the prognosis (Supplementary Fig. 4A and B). Consistent with the results in the database, we detected downregulated and upregulated expression of miR-326 and miR-330-5p in TNBC cells, respectively (Fig. 5B). Similarly, miR-326 expression in TNBC tissues was notably decreased (Fig. 5C). Patients with higher miR-326 expression also had longer overall survival times, according to the Kaplan‒Meier survival analysis (Fig. 5D). The expression level of circRPPH1 in TNBC tissues was then shown to be negatively linked with hsa-miR-326 by Pearson correlation analysis (Fig. 5E). Therefore, we predicted that circRPPH1 could bind to miR-326 in TNBC and thus function as a molecular sponge. We verified the effect of circRPPH1 knockdown or overexpression on the expression of miR-326. The outcomes demonstrated that circRPPH1 overexpression decreased miR-326 expression, whereas circRPPH1 knockdown had the reverse effect (Fig. 5F and G). According to the circMIR software of the ENCORI database, we created dual luciferase reporter plasmids with wild-type and mutant circRPPH1 sequences based on the area with the highest scores for circRPPH1 binding to miR-326. The luciferase activity of the wild-type plasmid was demonstrated to be decreased by the miR-326 mimic, but the mutant plasmid showed no apparent decrease (Supplementary Fig. 4C, Fig. 5H and I). CircRPPH1 and miR-326 were primarily found in the cytoplasm, according to FISH tests (Fig. 5J and K). Additionally, we conducted RNA pull-down experiments in TNBC cells using circRPPH1 probes that were biotin-labeled, and the outcomes revealed a substantial rise in the enrichment of circRPPH1 and miR-326 in the biotin-labeled circRPPH1 probe group (Fig. 5L and M). In conclusion, circRPPH1 may function as a molecular sponge and directly bind to miR-326.

### MiR-326 partially reverses the tumor-promoting effect of circRPPH1

**Fig. 6:**
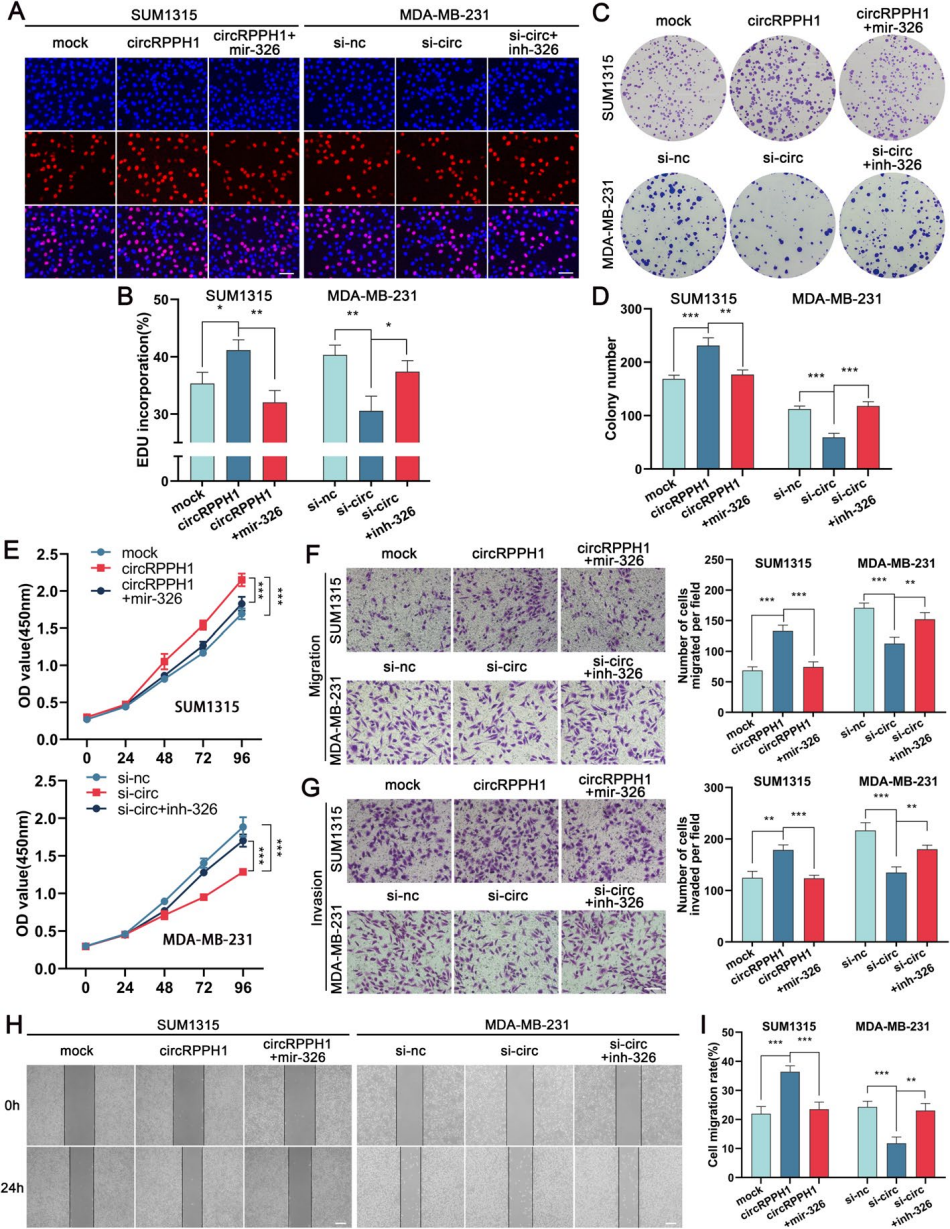
miR-326 partially reverses the effects of circRPPH1 in promoting TNBC cell proliferation, invasion and migration. **a-e** Detection of TNBC cell proliferation by EdU (magnification, × 200, scale bar, 50 μm), colony formation and cck8 assay, respectively, after transfection or co-transfection with circRPPH1 overexpression vector, si-circRPPH1, miRNA mimics or inhibitors after proliferative capacity of TNBC cells. **f-i** Transwell invasion (magnification, × 100, scale bar, 100 μm), transwell migration (magnification, × 100, scale bar, 100 μm), and wound healing (magnification, × 40, scale bar, 100 μm) were assayed after transfection or co-transfection with circRPPH1 overexpression vector, si-circRPPH1, miRNA mimics or inhibitors after invasion and migration ability of TNBC cells

To learn more about how miR-326 and circRPPH1 interact, we performed rescue experiments using miR-326 mimics or repressors with si-circ or the overexpression vector circRPPH1. Colony formation, EdU and CCK-8 assays indicated that miR-326 mimics blocked the impact of circRPPH1 overexpression on TNBC cell growth, whereas miR-326 inhibitors restored the effect of circRPPH1 knockdown on TNBC cell proliferation (Fig. 6A-E). Furthermore, regarding the impact of miR-326 on TNBC cell invasion and migration, we found that its promotion by overexpression of circRPPH1 could be inhibited by miR-326 mimics, whereas its inhibition by knockdown of circRPPH1 was partially reversed by miR-326 inhibitors (Fig. 6F-I). The above experiments demonstrated that miR-326 functions as an oncogenic factor to counteract circRPPH1's pro-oncogenic activities.

### *CircRPPH1 stimulates the FAK/PI3K/AKT pathway *via* the miR-326/ITGA5 axis*

**Fig. 7:**
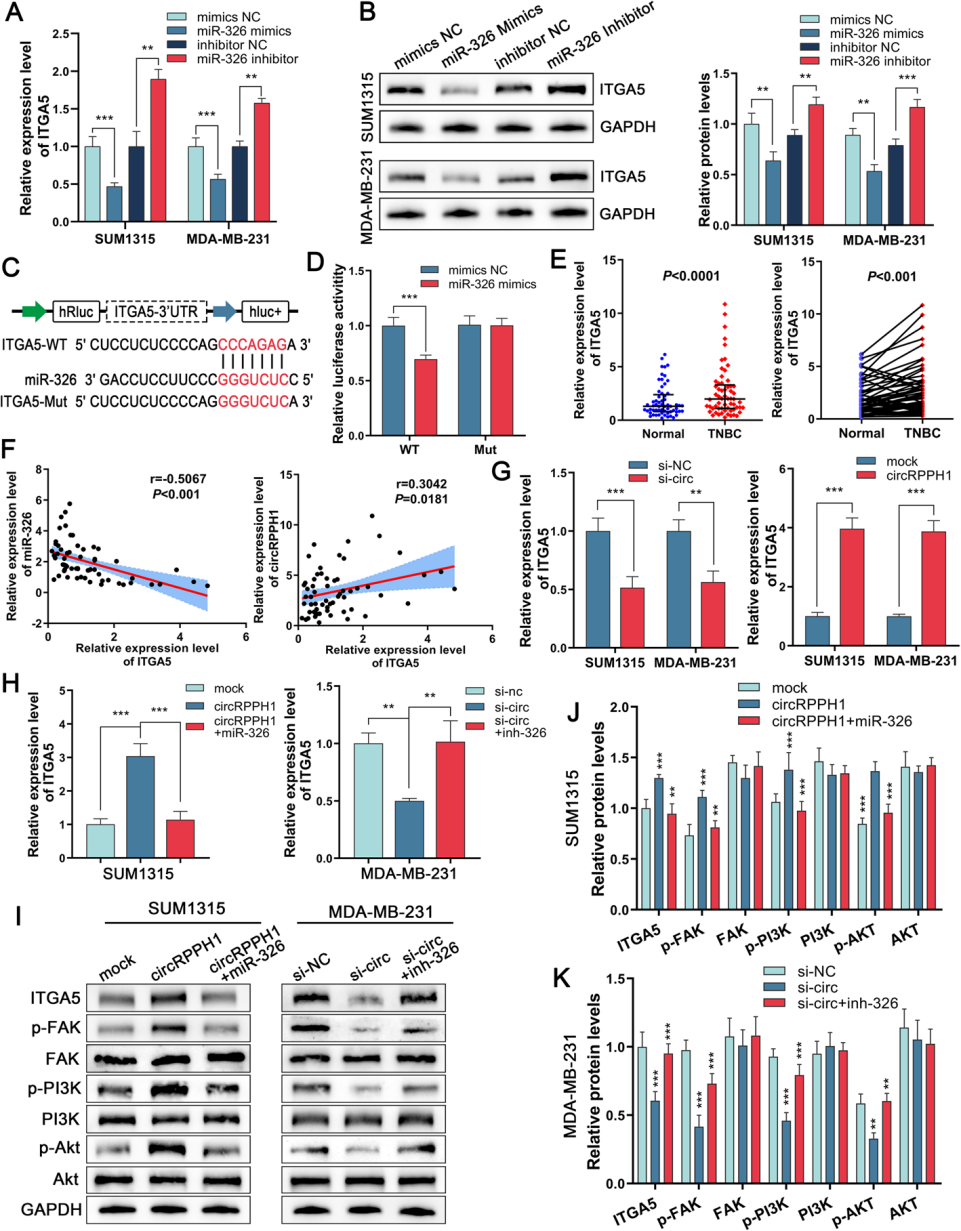
ITGA5 is a direct target of miR-326. circRPPH1 can act as a molecular sponge for miR-326 to regulate ITGA5 and activate the FAK/PI3K/Akt pathway. **a** and **b** The relative expression of ITGA5 was detected by qRT-PCR and western blot after transfection with miR-326 mimics and miR-326 inhibitor, respectively. **c** Schematic representation of luciferase reporter vectors containing wild-type (WT) and mutant (Mut) ITGA5 3'-UTR sequences. **d** Relative luciferase activity was determined after co-transfection of 293 T cells with ITGA5 3'-UTR WT/Mut luciferase reporter vectors and miR-326 mimics or negative controls, respectively. **e** Relative expression of ITGA5 was detected in 60 pairs of TNBC tissues and normal breast tissues. **f** Pearson correlation analysis showed that ITGA5 was negatively correlated with miR-326 and positively correlated with circRPPH1 in 60 pairs of TNBC tissues and normal breast tissues. **g** Relative expression of ITGA5 after overexpression or knockdown of circRPPH1 in TNBC cells. **h** and **i** Relative expression of ITGA5 after transfection or co-transfection of circRPPH1 overexpression vector, si-circRPPH1, miRNA mimics or inhibitors in TNBC cells. **j** and **k** Western blot detection of ITGA5 and FAK/PI3K/Akt pathway protein expression levels in TNBC cells after transfection or co-transfection with circRPPH1 overexpression vector, si-circRPPH1, miRNA mimics or inhibitors

It is understood that by attaching to the pre-mRNAs’ 3'-UTR to degrade them, miRNAs can regulate downstream target genes to perform their biological actions. To improve the circRPPH1/miR-326 axis and explore its downstream regulatory mechanism, we used the miRTarBase database (https://mirtarbase.cuhk.edu.cn/), miRBD database (https://mirdb.org/) and TargetScan database (https://www.targetscan.org/) to predict that ITGA5 may be the downstream gene of miR-326. With the use of Western blot and qRT‒PCR, we were able to show that miR-326 mimic transfection of SUM1315 cells and MDA-MB-231 cells reduced ITGA5 expression while increasing ITGA5 expression when miR-326 inhibitor was used (Fig. 7A and B). To confirm the interaction between ITGA5 and miR-326, we created dual luciferase reporter plasmids with wild-type and mutant ITGA5 3'UTRs. According to the findings, transfection of miR-326 mimics notably decreased the luciferase activity of wild-type plasmids but had no significant effect on mutant plasmids (Fig. 7C and D). We then verified that ITGA5 was upregulated in TNBC tissue (Fig. 7E). A Pearson correlation study revealed a positive association between circRPPH1 and ITGA5, but miR-326 and ITGA5 expression levels were found to be negatively correlated (Fig. 7F). Then, we detected ITGA5 expression after knockdown or overexpression of circRPPH1 in SUM1315 and MDA-MB-231 cells. The findings demonstrated that overexpression of circRPPH1 improved ITGA5 expression, while the opposite was observed for knockdown (Fig. 7G). Afterward, we discovered through rescue experiments that miR-326 mimics or inhibitors may undo the modifications in ITGA5 expression brought on by circRPPH1 overexpression or knockdown (Fig. 7H). According to KEGG pathway analysis, ITGA5 is a related gene in the PI3K/AKT pathway. As a result, we analyzed ITGA5 and its downstream genes' expression after upregulation or downregulation of circRPPH1 in TNBC cells. In TNBC cell lines, we discovered that overexpressing circRPPH1 significantly increased the expression of ITGA5, p-FAK, p-PI3K, and p-AKT, but transfection with si-circRPPH1 had the opposite effect. Additionally, miR-326 mimics or inhibitors might counteract these effects (Fig. 7I–K). These findings imply that miR-326/ITGA5 axis-mediated activation of the FAK/PI3K/AKT pathway by circRPPH1 promotes the aggressiveness of TNBC cells.

### *CircRPPH1 facilitates TNBC cell growth and metastasis *in vivo

**Fig. 8:**
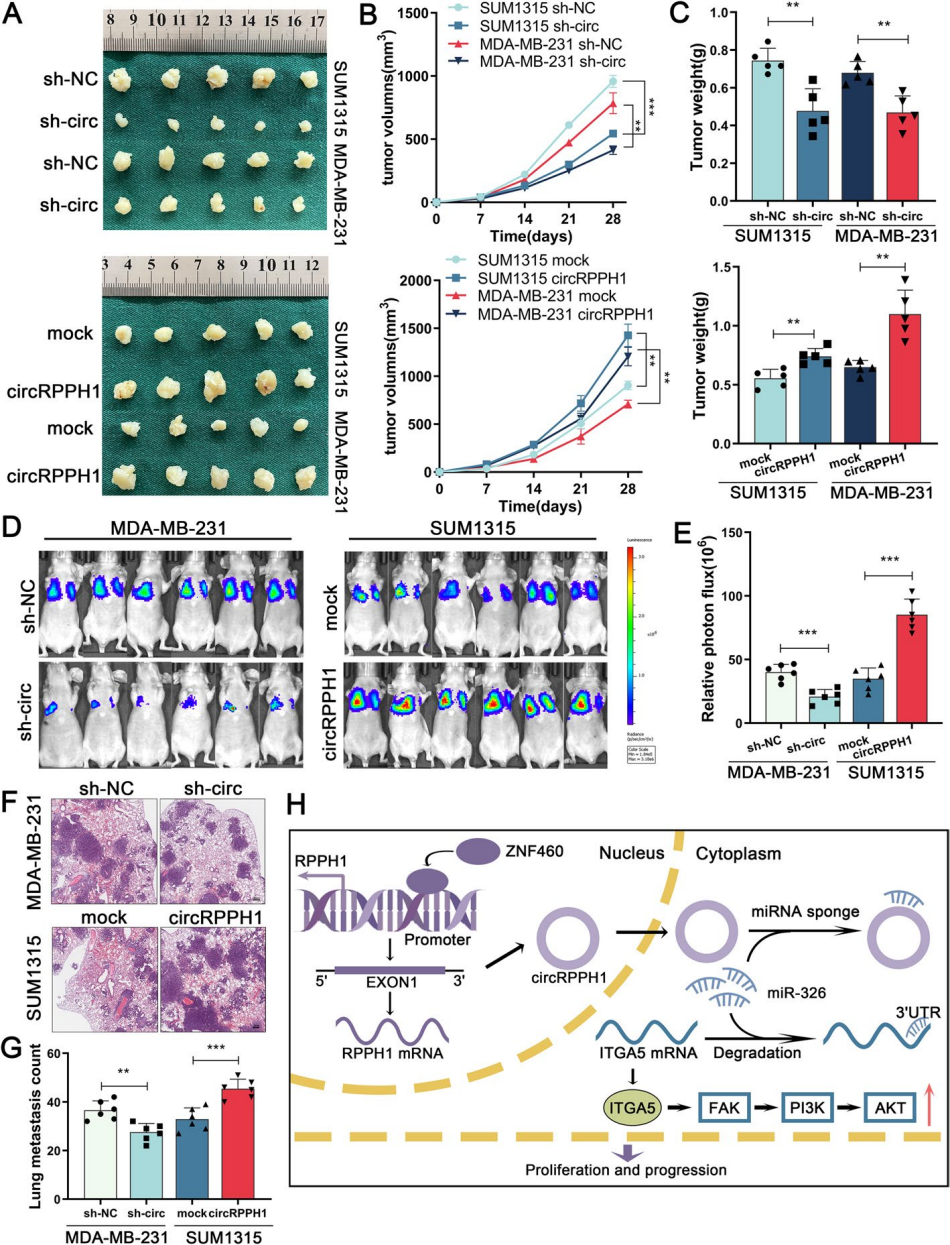
circRPPH1 promotes TNBC onset and progression in vivo. **a** The specified tumor cells were injected subcutaneously into nude mice, and xenograft tumors were collected 28 days later. **b** and **c** Tumor volumes were measured and counted weekly. **d** and **e** Bioluminescence imaging and quantification of lung metastases after tumor cells were injected into the tail vein of nude mice. **f** and **g** Representative images of lung metastases in mice and quantification of H&E staining (scale bar, 200 μm). **h** Schematic illustration of circRPPH1's cancer-promoting mechanisms in TNBC cells

We constructed TNBC cell lines that stably knocked down or overexpressed circRPPH1 to verify the biological function of circRPPH1 in vivo. Then, we injected the above cell lines into nude mice subcutaneously and observed for tumor growth and size. In vivo experiments showed that tumor formation was delayed after knockdown of circRPPH1, and the time to tumor formation in all nude mice was increased from within 9 days to within 18 days or 21 days, respectively. After inoculating circRPPH1-overexpressing cell lines, the tumorigenesis rate increased from 2/5 and 1/5 to 4/5 and 5/5 respectively on day 3, and all tumors were formed within 6 days (Supplementary Fig. 5A). Tumor size and weight were significantly reduced in the circRPPH1 knockdown group compared to the control group, whereas the results were reversed after overexpression of circRPPH1 (Fig. 8A-C). We subsequently conducted immunohistochemistry tests, which demonstrated that the circRPPH1 knockdown group's xenograft tumor tissues had significantly lower levels of both Ki-67 and ITGA5, but Ki-67 and ITGA5 were significantly upregulated in the circRPPH1 overexpression group (Supplementary Fig. 5B-E). We injected TNBC cells infected with luciferase plasmid into the tail vein of nude mice to examine the impact of circRPPH1 on metastasis. Bioluminescence imaging and H&E staining assays demonstrated that the quantity and size of lung metastases were considerably reduced following circRPPH1 knockdown, while the effects of circRPPH1 overexpression were the opposite (Fig. 8D-G). Western blotting of tumor tissues revealed that the ITGA5 expression level in the circRPPH1 overexpression group was much higher than that in the control group, while the opposite result was observed when circRPPH1 was knocked down (Supplementary Fig. 5F and G). The above results imply that circRPPH1 induces ITGA5 to facilitate TNBC cell growth and metastasis in vivo.

## Discussion

CircRNAs are circular molecules produced by backward splicing of pre-mRNAs and are found primarily in the cytoplasm. Because they are covalently linked at the head and tail, circRNAs are difficult for RNA enzymes to destroy. With the advancement of sequencing in recent years, our understanding of circRNAs has also increased. There is increasing evidence that aberrant circRNA expression can be vital for the development of cancer. Circular RNAs can act as sponges to regulate the activity of miRNAs [30]. CircRNAs may function as regulators in exosomes or binding proteins [31, 32]. Furthermore, several investigations have demonstrated that circRNAs encode functional peptides that influence cancer pathogenesis and progression [33]. However, only a small fraction of reported circRNAs have been associated with TNBC. Therefore, it is necessary to identify new circRNAs that can be used as therapeutic or diagnostic targets for TNBC.

In our study, by analyzing sequencing data of TNBC tissues and validation by qRT‒PCR, we screened out circRNA hsa-circ-000166 (called circRPPH1 in this study), which is upregulated in TNBC tissues and cells from the RPPH1 gene. In addition, by analyzing the clinical data and follow-up information collected by our investigators, we discovered a correlation between circRPPH1 expression and TNM stage. An adverse prognosis is suggested with high circRPPH1 expression. The above results indicate that circRPPH1 may become a new therapeutic or diagnostic target for TNBC. Then, to confirm its biological role, we carried out a number of functional assays. Experiments both in vitro and in vivo revealed that overexpressing circRPPH1 increased TNBC cell invasion and growth and accelerated tumor development and metastasis. Knockdown of circRPPH1 had the opposite effect. We then focused on the mechanisms by which circRPPH1 promotes the malignant biological behavior of TNBC. We found that the transcription factor ZNF460 could mediate the biogenesis of circRPPH1. Additionally, circRPPH1 binds to miR-326 and prohibits it from degrading ITGA5; thus, the FAK/PI3K/AKT pathway is activated, resulting in the formation and growth of TNBC. According to these conclusions, circRPPH1 could serve as a useful diagnostic tool and treatment target for TNBC.

Recent data suggest that backsplicing requires the spliceosome mechanism and that the regulation of circRNA formation is dependent on cis-acting elements and trans-acting factors [34]. We identified the binding location of ZNF460 to the RPPH1 gene promoter using bioinformatics predictions, which were then verified using dual luciferase reporter gene vectors and ChIP assays. We demonstrated by qRT‒PCR that overexpression of ZNF460 promoted circRPPH1 expression, while knockdown of ZNF460 inhibited circRPPH1 expression, suggesting that ZNF460 may promote transcription of the RPPH1 gene and production of circRPPH1.

MiRNAs directly target the mRNA 3'-UTR through base pairing and are able to regulate gene expression post-transcriptionally [35]. CircRNAs can interact with miRNAs, prevent their degradation of downstream genes, and affect cancer development [36]. For instance, hsa_circRNA_104348 influences the degradation of RTKN2 and consequently the development of hepatocellular carcinoma by serving as a sponge for miR-187-3p [37]. Additionally, circRNA cRAPGEF5 blocks the miR-27a-3p/TXNIP pathway, which prevents renal cell carcinoma from growing and metastasizing[38]. In addition, circRNF20, as a sponge of miR-487a, inhibits its degradation of hypoxia-inducing factor-1α (HIF-1α), thereby promoting breast cancer tumorigenesis and the Warburg effect[39]. In this research, we found that circRPPH1 may bind to miR-326 as a molecular sponge through bioinformatics analysis. There has already been evidence that miR-326 can have tumor suppressor effects in a variety of cancers. For example, miR-326 can bind to the Sp1 3'-UTR and indirectly decrease the expression of KLF3, thereby inhibiting lung cancer progression [40]. In addition, miR-326 can bind and inhibit the expression of the oncogene SMO to suppress the malignant biological behavior of glioma [41]. The TCGA dataset revealed that miR-326 is significantly downregulated in TNBC tissues and positively correlates with patient survival. Both circRPPH1 and miR-326 were found to be localized to the cytoplasm by FISH assay. Additionally, dual luciferase reporter gene assays and RNA probe pull-down tests proved that circRPPH1 could bind to miR-326. We next confirmed that miR-326 expression was downregulated and negatively associated with circRPPH1 in TNBC tissues and cells. Additionally, poor prognosis was indicated by low levels of miR-326 expression, which was consistent with the TCGA database. We carried out a number of rescue experiments to further confirm the biological role of miR-326. The findings revealed that miR-326 could act as an oncogene to partially reverse the pro-carcinogenic effects of circRPPH1.

According to the ceRNA theory, circRNAs serve as molecular sponges for miRNAs to influence downstream target gene expression [42]. We used the miRDB, TargetScan and miRTarBase websites for bioinformatics studies to refine the circRPPH1/miR-326 axis. The results showed that ITGA5 in the integrin family is one of the predicted targets of miR-326. Dysregulation of ITGA5 expression leads to cancer development and progression. For example, the high expression of ITGA5, a breast cancer bone metastasis-related gene, predicts a higher risk of bone metastasis and poor prognosis [43]. Further, miR-205 inhibits TNBC cell proliferation and metastasis by suppressing ITGA5 expression [44]. We then found that ITGA5 expression was reduced by upregulation of miR-326, while miR-326 downregulation had the opposite effect. It was determined that miR-326 targets the 3'-UTR of ITGA5 via dual luciferase reporter gene experiments. ITGA5 and FAK are upstream regulators of PI3K and Akt signaling. FAK is an important kinase in integrin signal transduction and is necessary to activate both PI3K and AKT [45, 46]. A study revealed that miR-326 can target ITGA5 and inhibit the FAK/Src pathway in TNBC and reverse fibronectin (FN1)-driven chemoresistance [47]. In addition, PI3K/AKT has kinase activity, and its activation by phosphorylation regulates cell proliferation, growth and metabolism [48]. The overexpression and phosphorylated activation of PI3K/AKT is a significant factor leading to the development of a variety of cancers [49]. For example, lack of miR-148a-3p in hepatic stellate cell exosomes reduces ITGA5 degradation and promotes tumor progression in HCC via the PI3K/AKT axis [28]. In this study, we verified that ITGA5 was upregulated in TNBC tissues and cells, and its expression was adversely related to miR-326 and contrasted with circRPPH1. In addition, downstream phosphorylation of the integrin kinase FAK and the signaling molecule PI3K/AKT was also increased in TNBC cells overexpressing circRPPH1, whereas activation of FAK and PI3K/AKT was reduced in circRPPH1 knockdown TNBC cells. MiR-326 mimics or inhibitors also partially reversed these effects. The above results support the presumption that in TNBC, circRPPH1 binds to miR-326 and limits the degradation of ITGA5, which activates the FAK/PI3K/AKT pathway.

## Conclusions

In summary, we discovered a new circRNA called circRPPH1 that is considerably overexpressed in TNBC and indicates a poor prognosis. Our study demonstrated that circRPPH1 could bind to miR-326 to control the expression of ITGA5 and activate the FAK/PI3K/AKT pathway, resulting in the development of TNBC. Therefore, circRPPH1 may provide a useful diagnostic tool and potential treatment target for TNBC.

### Supplementary Information

**Additional file 1.**

**Additional file 2.**

**Additional file 3.**

**Additional file 4.**

## Data Availability

All the data used in the current study are available from the corresponding authors upon reasonable request.

## Abbreviation

circRNAs: Circular RNAs
ER: Estrogen receptor
PR: Progesterone receptor
HER2: Human epidermal growth factor receptor 2
miRNAs: MicroRNAs
3'-UTR: 3' Untranslated region
ceRNA: Competing endogenous RNA
ncRNA: Non-coding RNA
ROC: Receiver operating characteristic curves
