# Supplementary material for: ZNF460-mediated circRPPH1 promotes TNBC progression through ITGA5-induced FAK/PI3K/AKT activation in a ceRNA manner
Source: Mol Cancer. 2024 Feb 14;23:33. doi: 10.1186/s12943-024-01944-w (PMC10865535; doi:10.1186/s12943-024-01944-w)
Supplement: Supplementary file 1 — Additional file 1. [file 12943_2024_1944_MOESM1_ESM.doc]

**Table S1 Sequences of siRNAs and shRNAs used in this study**

| **Definition** | **sequences** |
| --- | --- |
| si-NC | 5'-UUCUCCGAACGUGUCACGUTT-3' |
| si-circ1 | 5'-GGUGAGGUGAGUUCCCAGAGA-3' |
| si-circ2 | 5'-AUGGCUGAGGUGAGGUGAGUU-3' |
| si-circ3 | 5'-CUGAGGUGAGGUGAGUUCCCA-3' |
| sh-NC | 5’-CCGGATATTGCATTTGATACTCGACTCGAGTCGAGTATCAAATGCAATAT-3’ |
| sh1-ZNF460 | 5ʹ-CACCAAGTATTCCTGCATTTCAGATCTCGAGATCTGAAATGCAGGAATAC-3’ |
| sh2-ZNF460 | 5ʹ-CACCAATTCTCATTAAACATTTCCTCTCGAGAGGAAATGTTTAATGAGAA-3’ |
| sh3-ZNF460 | 5ʹ-CACCATATTAAAAAACTATTTCGCTCTCGAGAGCGAAATAGTTTTTTAATA-3’ |
