# Supplementary material for: ZNF460-mediated circRPPH1 promotes TNBC progression through ITGA5-induced FAK/PI3K/AKT activation in a ceRNA manner
Source: Mol Cancer. 2024 Feb 14;23:33. doi: 10.1186/s12943-024-01944-w (PMC10865535; doi:10.1186/s12943-024-01944-w)
Supplement: Supplementary file 2 — Additional file 2. [file 12943_2024_1944_MOESM2_ESM.doc]

**Supplemental methods**

**RNA isolation, nucleus-cytoplasmic separation, RNAase R and qRT-PCR**

Total RNA was isolated using TRIzol reagent (Invitrogen, CA, USA) according to instructions, and RNA concentration and purity were detected by NanoDrop spectrophotometer (ND-100, Thermo). Nucleus and cytoplasmic RNAs from TNBC cells were isolated using PARIS™ Kit (Invitrogen, Carlsbad, CA, USA).For the RNase R assay, TNBC cells were treated with 3 U/μg RNase R (Beyotime, Shanghai, China) for 10, 20, 30, and 40 minutes at 37°C. After determining the RNA concentration, the reverse transcription reaction was conducted with HiScript II 1st Strand cDNA Synthesis Kit (+gDNA wiper) (Vazyme Biotech, Nanjing, China). Quantitative real-time fluorescence PCR (qRT-PCR) was then detected using SYBR Green reagent (Vazyme Biotech, Nanjing, China). For miRNAs, reverse transcription was performed using miRNA 1st Strand cDNA Synthesis Kit (by stem-loop) (Vazyme Biotech, Nanjing, China). Real-time fluorescence quantitative PCR (qRT-PCR) was performed using miRNA Universal SYBR qPCR Master Mix (Vazyme Biotech, Nanjing, China). The endogenous reference for circRNAs and mRNAs is GAPDH, and the endogenous reference for miRNAs is U6. Expression results were detected on an ABI StepOne Plus qRT-PCR machine and system. The 2ΔΔCt method was used to calculate the relative expression.

| **Gene** | **Primer sequences** |
| --- | --- |
| GAPDH | F: 5'- GAAGGTGAAGGTCGGAGTC-3' |
|  | R: 5'- GAAGATGGTGATGGGATTTC-3' |
| U6 | F: 5'-CTCGCTTCGGCAGCACA-3' |
|  | R: 5'-AACGCTTCACGAATTTGCGT-3' |
|  | RT: 5'-AACGCTTCACGAATTTGCGT-3' |
| RPPH1 | F: 5'-AGCTTGGAACAGACTCACGG-3' |
|  | R: 5'-AATGGGCGGAGGAGAGTAGT-3' |
| circRPPH1  (divergent primers) | F: 5'- CTCCTTTGCCGGAGCTTG -3' |
|  | R: 5'- GGTCCACGGCATCTCCTG -3' |
| circRPPH1  (convergent primers) | F: 5'- GTTCCCAGAGAACGGGGCTC -3' |
|  | R: 5'- GTTCCAAGCTCCGGCAAAGG -3' |
| hsa-mir-326 | RT:5'-GTCGTATCCAGTGCAGGGTCCGAGGTATTCGCACTGGATACGACCTGGAG -3' |
|  | F: 5'- CGCCTCTGGGCCCTTC -3' |
|  | R: 5'- AGTGCAGGGTCCGAGGTATT -3' |
| ITGA5 | F: 5'- GTCGGGGGCTTCAACTTAGAC -3' |
|  | R: 5'- CCTGGCTGGCTGGTATTAGC -3' |
| ZNF460 | F: 5'- CTCATTCGACACTTCAACATCC -3' |
|  | R: 5'- GTGGATGCTAAAGTGTCGAATC -3' |

**Western blot**

Cells were treated with RIPA Lysis Buffer (Beyotime, Shanghai, China) and the concentration was determined by BCA Protein Assay Kit (Beyotime, Shanghai, China). Proteins were separated on SDS-PAGE gels and transferred to PVDF membranes (Millipore, Bedford, MA, USA). The membrane was blocked with QuickBlock™ Blocking Buffer for Western Blot (Beyotime, Shanghai, China), incubated with primary antibody at 4°C overnight, and then rinsed with TBST buffer three times for 10 min each. The membrane was incubated with the corresponding enzyme-conjugated secondary antibody and detected using Super ECL Plus (Proteinbio, Nanjing, China). The antibodies used in this study are shown in the table below.

| **Antibody** | **Company** | **Catalog number** |
| --- | --- | --- |
| anti-GAPDH | proteintech | 60004-1-Ig |
| anti-Mouse IgG(H+L) | proteintech | SA00001-1 |
| anti-Rabbit IgG(H+L) | Proteintech | SA00001-2 |
| anti-N-cadherin | Cell Signaling Technology | 13116 |
| anti-E-cadherin | Proteintech | 20874-1-AP |
| anti-Vimentin | Cell Signaling Technology | 5741 |
| anti-Bax | abmart | T40051 |
| anti-Bcl-2 | Cell Signaling Technology | 3498 |
| anti-Cleaved Caspase-3 | Cell Signaling Technology | 9661 |
| anti-Cyclin E1 | Proteintech | 11554-1-AP |
| anti-Cyclin D1 | Proteintech | 60186-1-Ig |
| anti-CDK4 | Cell Signaling Technology | 12790 |
| anti-ITGA5 | proteintech | 27224-1-AP |
| anti-ZNF460 | proteintech | 25299-1-AP |
| anti-p-AKT | abmart | T40067 |
| anti-AKT | abmart | T55561 |
| anti-p-PI3K | abcam | ab182651 |
| anti-PI3K | abcam | ab191606 |
| anti-p-FAK | Cell Signaling Technology | 3283 |
| anti-FAK | Cell Signaling Technology | 3285 |

**Fluorescence in situ hybridisation (FISH)**

To observe the co-localisation of circRPPH1 and hsa-miR-326 in TNBC tissues and cells, we designed a Cy3-labeled circRPPH1 probe (5'-CTCTGGGAACTCACCTCACCTCA-3') and a FITC-labeled hsa-miR-326 probe (5'-CTGGAGGAAGGGCCCAGAGG-3') at Geneseed (Guangzhou, China). Analyses were performed using a fluorescence in situ hybridisation kit according to the manufacturer's instructions. Cell nuclei were stained with DAPI (Beyotime, Shanghai, China). Images were captured under a fluorescence microscope (Leica, Wetzlar, Germany).

**Dual luciferase reporter assay**

The circRPPH1 and ITGA5 3'UTR sequences and their corresponding mutant versions were synthesised and subcloned into the psiCHECK™-2 vector (Promega, Madison, WI, USA), designated circRPPH1-Wt, circRPPH1-Mut, ITGA5 3'UTR-Wt and ITGA5 3'UTR-Mut, respectively. The corresponding plasmids and hsa-miR-326 mimic/miR-NC or hsa-miR-326 inhibitor/inh-NC were co-transfected into 293T cells using Lipofectamine 2000 (Invitrogen, Carlsbad, CA, USA). To construct luciferase reporter gene vectors containing the RPPH1 promoter, the full-length promoter containing wild-type(WT) and mutant(Mut) RPPH1 was cloned into pGL3-basic vector (Genecreate, Wuhan, China) and then co-transfected with ZNF460 overexpression vector or control vector into TNBC cells. After 48 hours of transfection, relative luciferase activity was assessed using a dual luciferase assay kit (Promega, Madison, WI, USA) according to the manufacturer's protocol.

**5-Ethyl-2'-deoxyuridine (EdU) assay, cell counting kit (CCK)-8 and colony formation assay**

DNA synthesis and cell proliferation of TNBC cells were assessed using the EdU assay kit (Beyotime, Shanghai, China) and CCK-8(DOJINDO, Japan), respectively, according to the manufacturer's protocols. For CCK-8, absorbance was measured at 450 nm using an automated microtome (BioTek, Winooski, VT, USA). In the colony formation assay, the treated cells inoculated in 6-well plates (500 cells/well) were incubated for 2 weeks, then the cells were fixed with methanol for 30 minutes, stained with crystal violet for 30 minutes and the number of cell colonies counted.

**Wound healing assay and transwell assay**

Cells from different treatment groups were inoculated into 6-well plates and incubated at 37°C until complete fusion. The tip of a 200 μL sterile pipette was passed through the cell monolayer to create a scratch and washed with PBS to remove surrounding cell debris. Images were captured using an inverted microscope (Olympus Optics Co., Ltd, Tokyo, Japan) (0 h) and the area of the scratch was marked. Serum-free medium was then added to the 6-well plate and incubated at 37°C for 24 h. The culture medium was then removed and the same area was photographed again after removing cell debris by washing with PBS. Cells were imaged using Image J software (NIH, USA) to analyse the wound width in the images. For the migration assay, the transwell chamber (Millipore, Billerica, MA, USA) was placed in a 24-well plate. The lower chamber was supplemented with 600 μL medium containing 10% fetal bovine serum, and the upper chamber was inoculated with 20,000 cells suspended in 200 μL serum-free medium. After 24 hours, the cells in the membrane were fixed with methanol and stained with crystal violet. Photographs were taken under an inverted fluorescence microscope for counting. To verify the invasive ability of the cells, 100 μL of Matrigel (BD Bioscience, San Jose, CA, USA) was added to the inner chamber and the above steps of cell inoculation were performed in the upper chamber.

**Cell cycle and apoptosis assays**

To investigate the effect of circRPPH1 on cell cycle and apoptosis, cells (4 × 10^5^/well) were plated in 6-well plates, incubated overnight and treated in different groups. For cell cycle analysis, cells were stained with propidium iodide (PI) using a cell cycle staining kit (MULTI SCIENCES, Hangzhou, China) according to the manufacturer's instructions. For apoptosis, cells were stained with Annexin V-FITC/PI Apoptosis Detection Kit (Vazyme, Nanjing, China) to double stain the cells. The proportion of different cell cycle phases and the percentage of early apoptotic cells were then analysed using a FACScan flow cytometer (BD, Franklin Lakes, New Jersey).

**Immunofluorescence (IF)**

TNBC cells with different treatments (2×10^4^ cells/well) were seeded on confocal plates (NEST, Wuxi, China) and incubated overnight, the cells were washed twice with PBS and fixed with 4% paraformaldehyde, then permeabilized with 0.2% Triton X-100. The cells were blocked with Immunol Staining Blocking Buffer (Beyotime, Shanghai, China) for 60 minutes and incubated with primary antibody at 4°C overnight, followed by incubation with fluorescein-conjugated secondary antibody at 4°C overnight. Finally, the cells were observed using a confocal microscope (Zeiss, Germany).

**Chromatin immunoprecipitation (ChIP)**

ChIP assay was performed using a ChIP kit (Bersin, Guangzhou, China). TNBC cells were cross-linked with 1% formaldehyde for 10 minutes and then neutralised with glycine. Cells were lysed and DNA fragments were fragmented by ultrasound until they were between 200 and 600 bp. The supernatants were incubated with ZNF460 antibody or IgG antibody overnight at 4°C and then with protein A/G magnetic beads for 30 min at room temperature. After washing and uncrosslinking, the enriched DNA was purified and detected by qRT-PCR.

**Biotin-coupled probe RNA pull-down assay**

To pull down miRNA by circRNA, biotinylated circRPPH1 probe (5'-CTCTGGGAACTCACCTCACCTCA-3') and control probe (5'-GCTCACACTTACTTCGACACCGC-3') were synthesized by RiboBio (Guangzhou, China). The probes were incubated with TNBC cell lysates overexpressing circRPPH1, and then streptavidin-coated magnetic beads (Thermo Fisher Scientific, MA, USA) were added to adsorb the biotin-coupled RNA complexes. The RNA complexes were eluted and extracted using Trizol. Enriched circRPPH1 and miR-326 were analysed by qRT-PCR and RT-PCR.
