# Supplementary material for: ZNF460-mediated circRPPH1 promotes TNBC progression through ITGA5-induced FAK/PI3K/AKT activation in a ceRNA manner
Source: Mol Cancer. 2024 Feb 14;23:33. doi: 10.1186/s12943-024-01944-w (PMC10865535; doi:10.1186/s12943-024-01944-w)
Supplement: Supplementary file 3 — Additional file 3. [file 12943_2024_1944_MOESM3_ESM.doc]

**
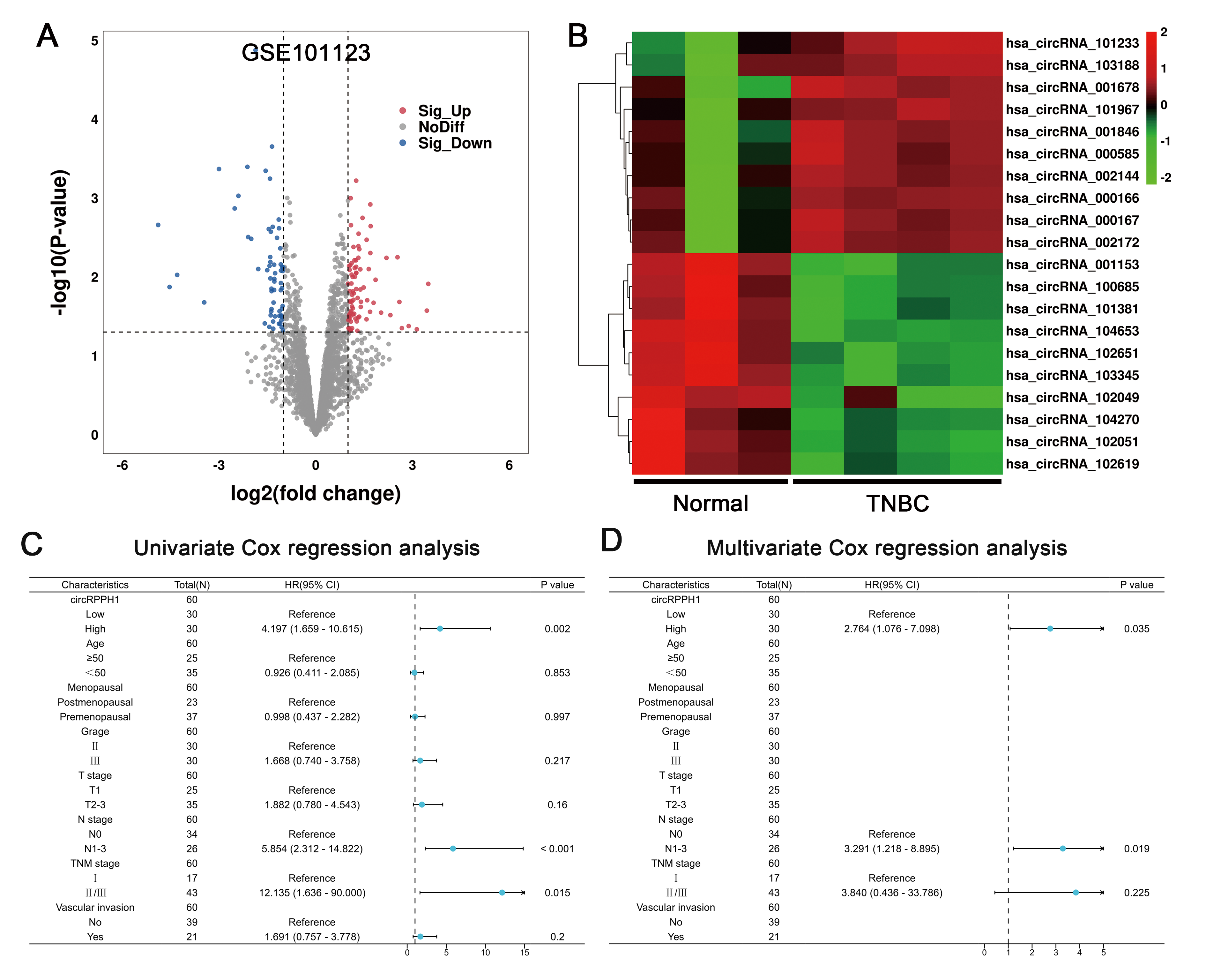
**

**Supplementary fig.1** Supplementary results of figure 1. **a** Volcano plot showing the identification of differentially expressed circRNAs between TNBC tissues and adjacent non-tumor tissues. red and blue dots represent statistically significant up- and down-regulated circRNAs, respectively. the cut-off value is set at |log2(fold change)|>1, P<0.05. **b** Clustered heatmap showing the top 10 up- and down-regulated circRNAs, respectively. Red and green bars indicate high and low expression, respectively. **c and d** Univariate and multivariate Cox regression analyses of circRPPH1 expression in TNBC and paracancerous tissues.


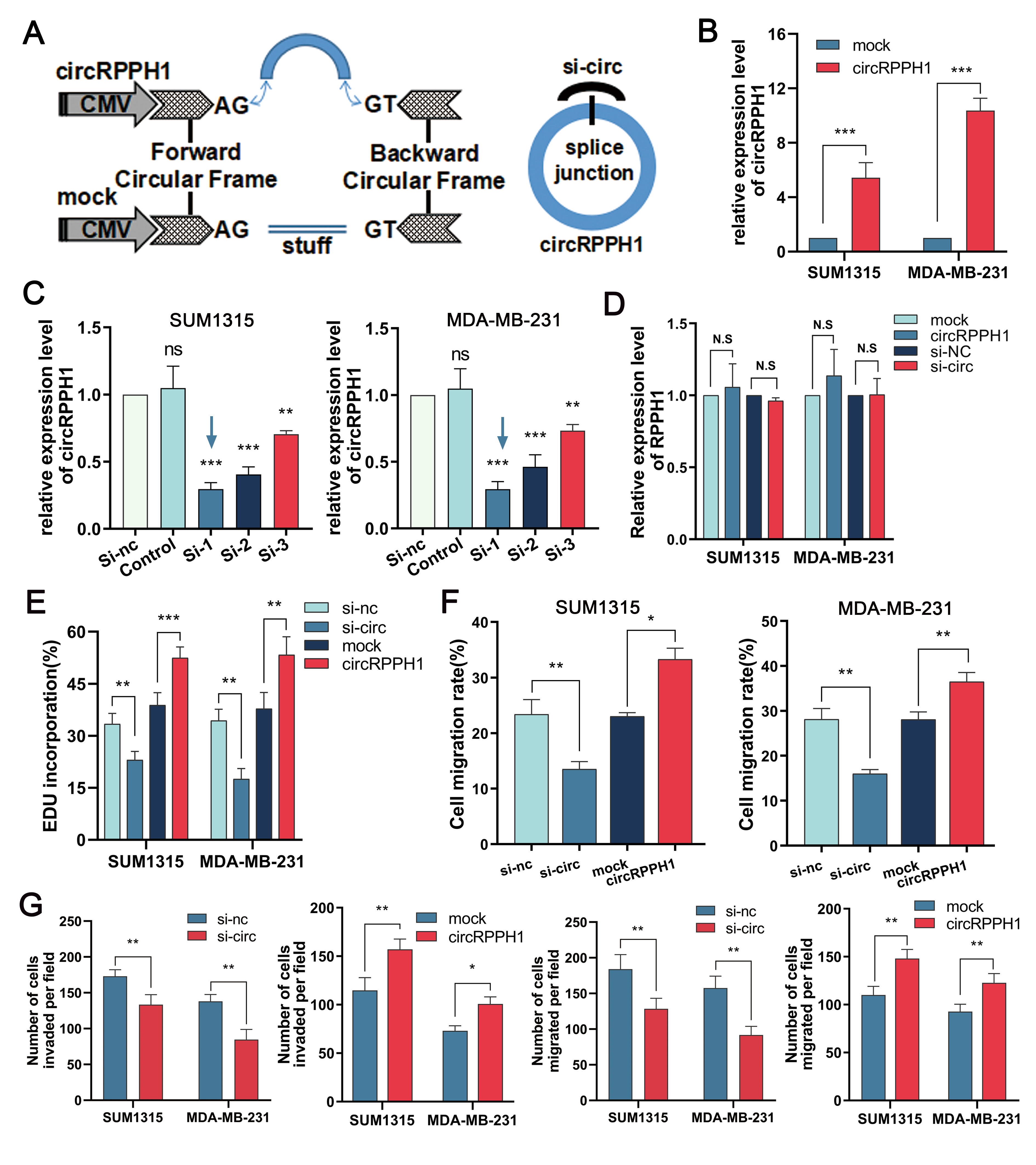


**Supplementary fig.2** Additional results of Figure 3. **a** Schematic diagram of circRPPH1 overexpression vector and siRNA. **b-d** Expression levels of circRPPH1 and RPPH1 in TNBC cells after transfection with circRPPH1 overexpression vector or siRNA. **e-g** Statistical results of Edu assay (Fig.3B), wound healing assay (Fig.3E), and transwell assay (Fig.3F).


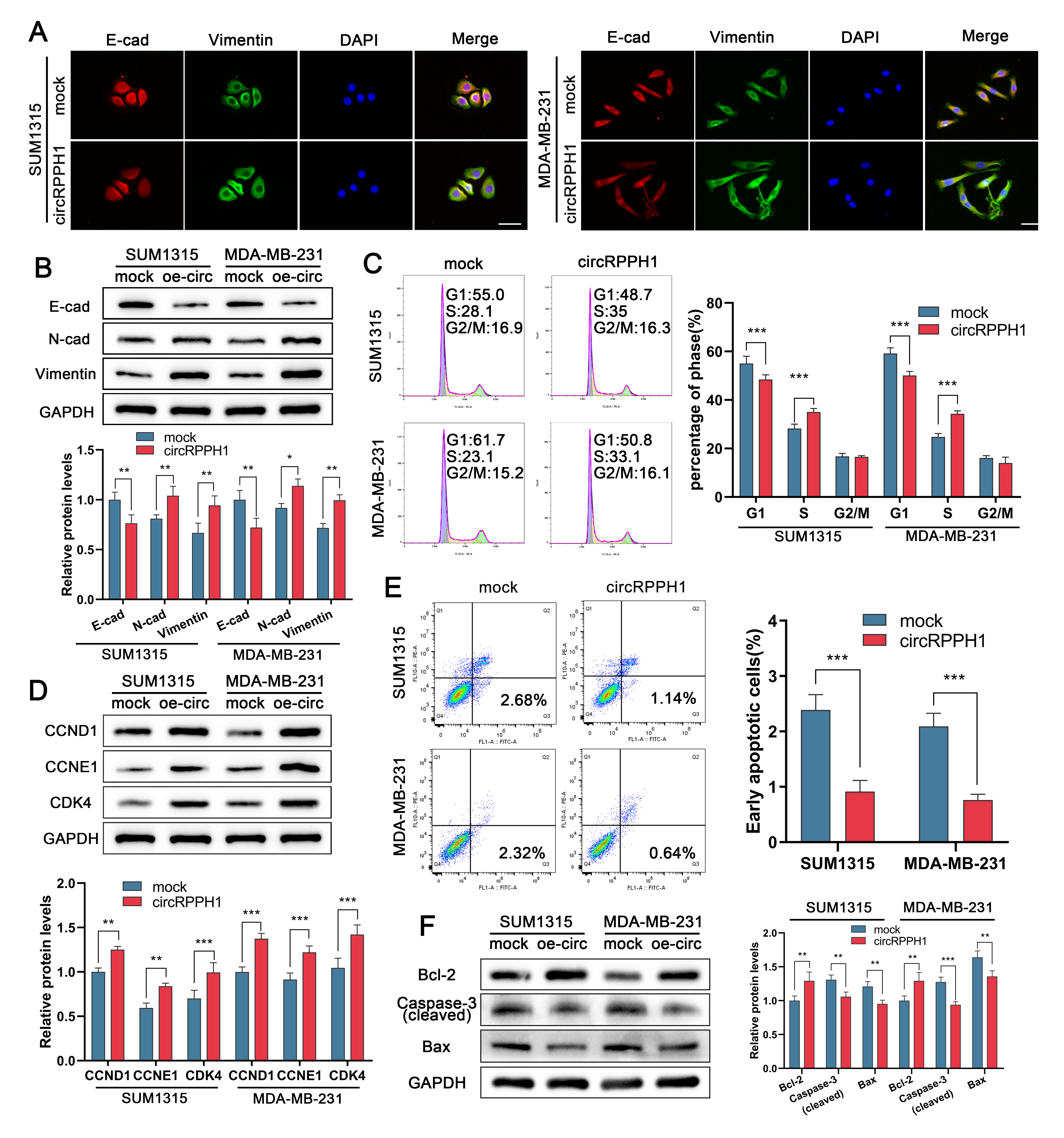


**Supplementary fig.3** Overexpression of circRPPH1 promotes EMT and regulates cell cycle and apoptosis in TNBC cells. **a** IF detection of EMT-related protein expression in TNBC cells after overexpression of circRPPH1 (magnification, ×200, scale bar, 50μm). **b** Western blot detection of EMT-related protein expression in TNBC cells after overexpression of circRPPH1. **c** Cell cycle analysis of TNBC cells transfected with circRPPH1 by flow cytometry. **d** Western blot detection of cell cycle-related protein expression after transfection of circRPPH1 in TNBC cells. **e** Early apoptosis rate of TNBC cells was detected by flow cytometry after overexpression of circRPPH1. **f** Western blot detection of apoptosis-related protein levels.


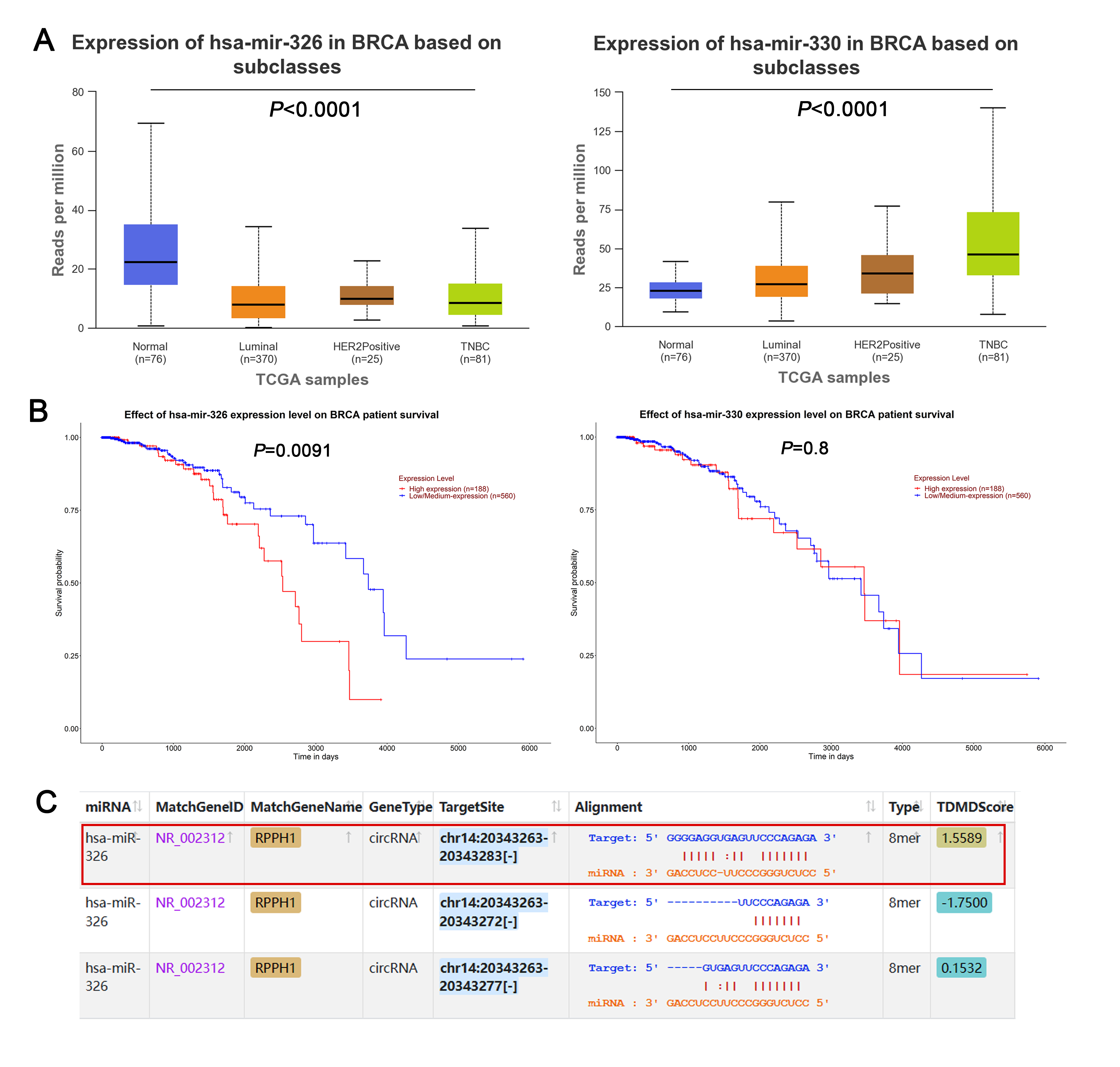


**Supplementary fig.4** Additional results of Figure 5. **a** TCGA database of has-mir-326 and has-mir-330 are down-regulated and up-regulated expression in TNBC, respectively. **b** Relationship between the expression levels of has-mir-326 as well as has-mir-330 in the TCGA database and overall survival of TNBC patients. **c** Prediction of circRPPH1 and miR-326 binding sites and corresponding scores in ENCORI database.

**
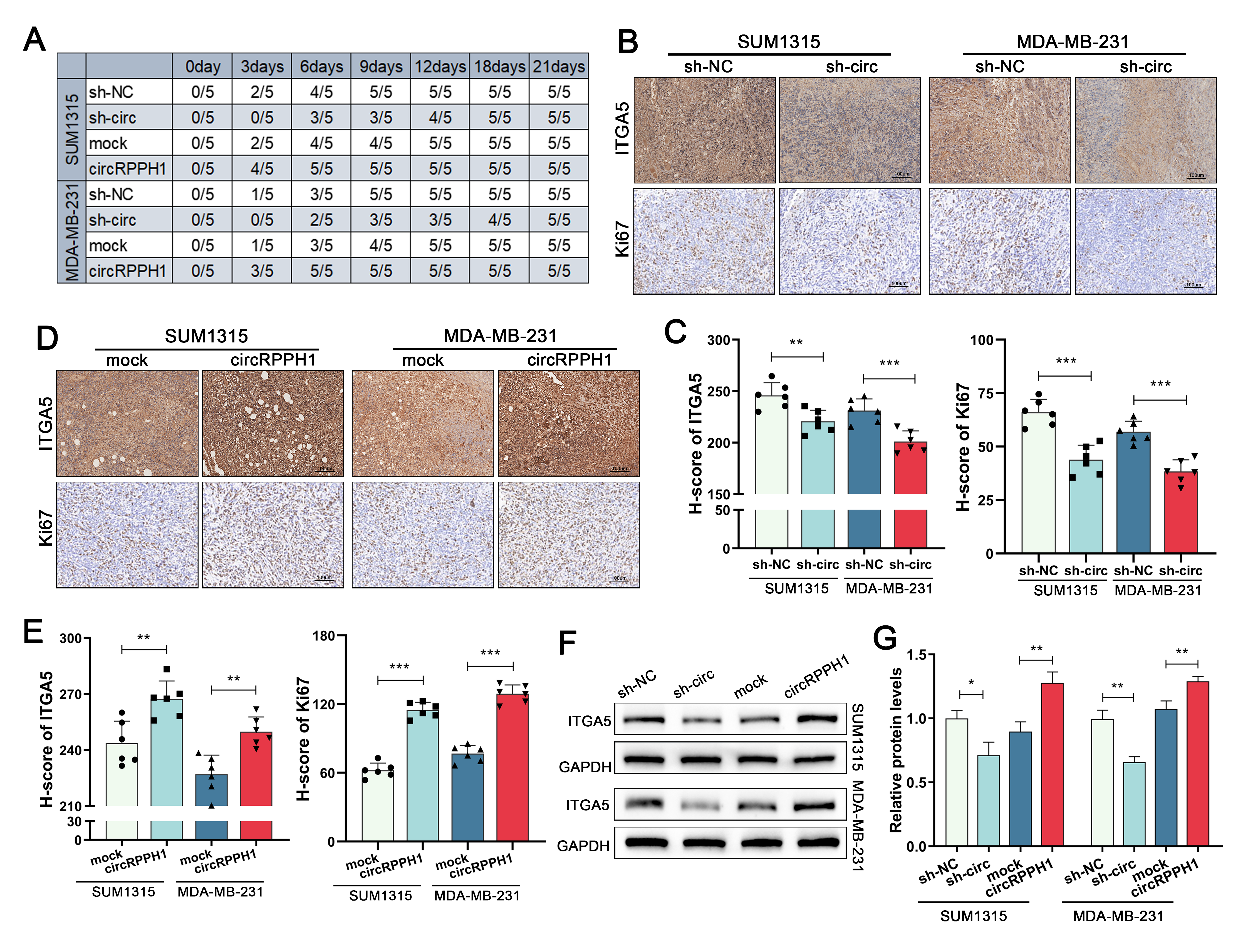
**

**Supplementary fig.5** Additional results of Figure 8. **a** Number of nude mice that formed tumors after injection of TNBC cell lines that knocked down or overexpressed circRPPH1. **b-e** IHC staining (scale bar, 100μm) and scoring of ITGA5 and Ki67 in xenograft tumor tissue. **f and g** Western blot analysis of ITGA5 protein expression levels in xenograft tumor tissues.
