# Supplementary figures and images for: ZNF460-mediated circRPPH1 promotes TNBC progression through ITGA5-induced FAK/PI3K/AKT activation in a ceRNA manner
Source: Mol Cancer. 2024 Feb 14;23:33. doi: 10.1186/s12943-024-01944-w (PMC10865535; doi:10.1186/s12943-024-01944-w)

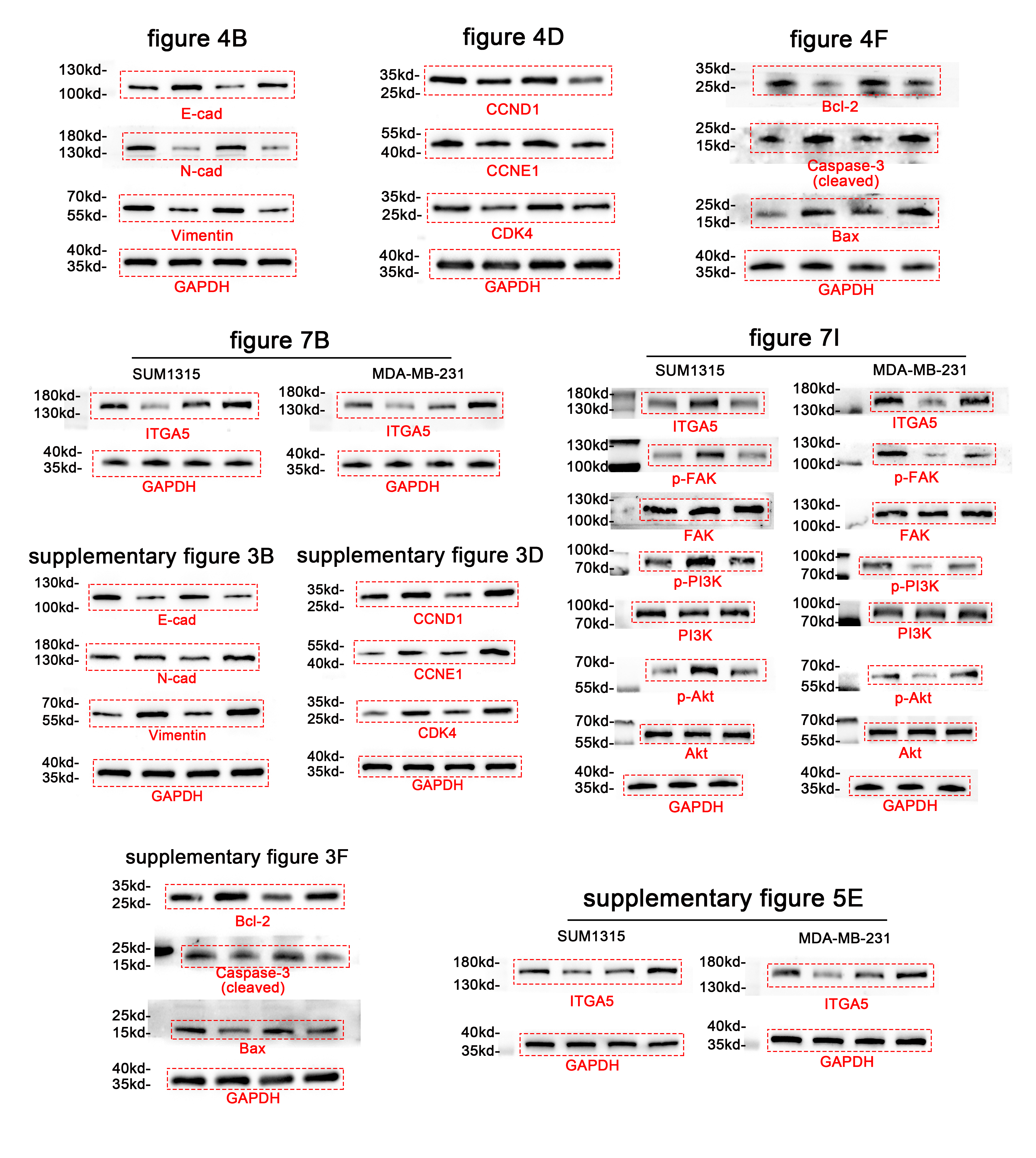

Supplement: Supplementary file 4 — Additional file 4. [file 12943_2024_1944_MOESM4_ESM.doc]
